# Immunoinformatics-Guided Computational Design and In Silico Validation of Multi-Epitope Vaccine Candidates Targeting Canine and Feline Parvoviruses

**DOI:** 10.3390/microorganisms14081721

**Published:** 2026-08-05

**Authors:** Nithyadevi Duraisamy, Abid Ullah Shah, Mohd Yasir Khan, Mohammed Cherkaoui, Maged Gomaa Hemida

**Affiliations:** 1 Department of Computer Science, College of Digital Engineering and Artificial Intelligence, Long Island University, Brooklyn, NY 11201, USA; nithyadevi.duraisamy@liu.edu (N.D.); mohd.yasirkhan@liu.edu (M.Y.K.); mohammed.cherkaoui@liu.edu (M.C.); 2 Department of Veterinary Biomedical Sciences, College of Veterinary Medicine, Long Island University, 720 Northern Boulevard, Brookville, NY 11548, USA; abidullah.shah@liu.edu

**Keywords:** feline, canine, parvovirus, multi-epitope vaccine, B-cell and T-cell epitope predictions, disulfide bond and normal mode analysis, molecular docking, molecular dynamic simulation, immune simulation, in silico cloning

## Abstract

Parvovirus infection causes severe diseases in both feline and canine species. It primarily affects adult cats and dogs but poses a higher risk to kittens and puppies. This virus is highly contagious and is easily transmitted through contaminated food, shared shelter environments, as well as the hands and clothing of people. The recovered species may continue to shed parvovirus in their feces for an extended period, leading to severe environmental contamination. There is no universal vaccine available that protects dogs and cats against parvovirus infections. The main goal of this study is to design a pan-parvovirus multiepitope-based vaccine that could be administered to dogs and cats. We utilized AI-machine learning-incorporated server tools such as IEDB and NetMHCpan to predict B-cell and T-cell epitopes. VaxiJen and ToxinPred were used to analyze immune characteristic features and docking with feline alleles using the HADDOCK server. Following this, the immune response and stability of the vaccine construct were confirmed with disulfide engineering, normal mode analysis, and molecular docking performed with toll-like receptors of both feline and canine (TLR4 and TLR5), and molecular dynamics simulation was performed for 10 ns. The triggered immune response was determined with immuno-simulation (ImmSim), and their activity in a biological environment was reinforced with in silico cloning. The B-cell epitopes (NS1-9, NS2-4, VP1-12 and VP2-9) predicted with the IEDB database were subjected to antigenicity prediction. MHC class I and IFN prediction and MHC class II and IL-4 prediction were performed with IEDB and NetMHCpan. The T-cell epitopes showed high binding affinities with the feline alleles. The final vaccine was designed by combining the top-ranked B-cell epitopes and T-cell epitopes, filtered for high antigenicity, non-allergic, non-toxic, and good solubility, and with the better binding affinity score of the structural and non-structural proteins (NS1, NS2, VP1, and VP2) of feline and canine parvoviruses through linkers and adjuvants. The disulfide bond prediction and normal mode analysis showed that our vaccine construct is stable and flexible. The molecular docking analysis was performed between the designed vaccine epitopes and the TLRs (TLR4–feline and TLR5–canine) with Biovia Discovery Studio using Zdock; it showed better binding interactions with a value of 22.26 (Zdock score), −47.409 (Zrank score) for feline and 16.54 (Zdock score), −134.295 (Zrank score) for canine. A pan-multi-epitope-based vaccine based on the two structural and non-structural proteins (NS1, NS2, VP1, and VP2) was designed and constructed to provide dual protection against parvovirus in both feline and canine species. The molecular docking and molecular dynamics simulation analysis showed higher binding affinities and stable conformations with canine (TLR5) and feline (TLR4) toll-like receptors. Although computational analysis supports the prediction of top-ranked epitopes and their immunogenic properties with greater precision, further experimental validation is required before they can be used against these viruses.

## 1. Introduction

Parvovirus is primarily characterized by non-suppurative myocarditis and acute hemorrhagic enteritis. It is a highly contagious and potentially life-threatening disease affecting feline and canine species of all ages, especially kittens and puppies [1]. Since both species behave quite similarly, the parvovirus originally evolved from feline and began infecting the canine species with slight modifications [2,3]. The main disadvantage is that the virus becomes infectious during the incubation period, before clinical signs become evident, making it difficult to differentiate infected animals from healthy ones. This is why animals can enter or leave the shelter healthy, even though they are affected. The existing way of measuring is through parvo diagnosis with the IDEXX brand SNAP test, though positive results should be considered even if the animals are vaccinated. The strains of parvovirus are not harder to diagnose or prevent, which could be detected with in-house fecal ELISA tests.

The conventional vaccines, such as live attenuated and inactivated vaccines, have been extensively used to protect animals and birds from many viral diseases with few limitations. Those vaccines do not have intact pathogens, eliminating the risk of viral shedding and avoiding allergic reactions caused by irrelevant antigenic components. Feline parvovirus (FPV) and canine parvovirus (CPV) do not mutate rapidly the way influenza viruses and other RNA viruses do; thus, the available vaccine in the market produced by major manufacturers provides excellent immunity to all variants [4,5]. This protection is not enhanced by multiple vaccinations, as young animals receive only a limited number of vaccine doses, making them more susceptible to these viruses [6]. Erin et al. developed modified live FPV (MLV) vaccines, and their results showed induced immunity in kittens compared to inactivated vaccines [7]. Another scientist named Wang et al. developed a vaccine with FPV virus forming them like particles using a recombinant baculovirus system, producing strong immunogenicity and protection against FPV in cats [8]. Though there are more effective vaccines, animals still lose their lives to these diseases due to misconceptions among pet owners and even some veterinary professionals. Overvaccination raises concerns among them; they choose to vaccinate less frequently or not vaccinate at all.

Indeed, the American Veterinary Medical Association’s handout (2010) stated that these viruses were once a leading cause of death in cats and dogs but are now an uncommon cause of disease, largely due to the availability and widespread use of highly effective vaccines. Some of these vaccines are outdated and were prepared a long time ago; thus, the viral strains used in these vaccines do not match the currently circulating field strains, which hamper the success of these vaccines. Another type of vaccine is the epitope vaccine, a subtype of subunit vaccine composed of multiple neutralizing antigenic epitopes, resulting in the production of higher levels of antibodies. Unlike traditional vaccines, these vaccines not only induce immune responses in hosts but also exclude the pathogen itself, which prevents viral transmission or mutations, resulting in enhanced safety [9]. The purpose and importance of developing a new multiepitope vaccine format are to address the potential challenges of ongoing viral evolution and to provide a more flexible and rapidly adaptable vaccine design approach. Additionally, this vaccine construct compiles CTL, HTL, and B-cell epitopes from both FPV and CPV, which helps enhance cross-protective immune coverage. 

The sizes of CPV and FPV are approximately 25 nm, and its genome has two open reading frames (ORFs). The first ORF has two non-structural proteins (NS1 and NS2), while the second ORF has two structural proteins (VP1 and VP2). In this, VP2 is the most critical, as it induces antibody production, making it a better candidate for vaccine design [6]. Many researchers have used this structural protein for developing genetically engineered vaccines and have been experimentally validated or predicted as potential antigenic sites. The mapping of these antigenic epitopes is very important, and current methods for evaluating these epitopes are performed through chemical cleavage or enzymatic digestion, X-ray crystallography, nuclear magnetic resonance (NMR) analysis, and computational approaches [10,11].

The emergence of new artificial intelligence tools, particularly machine learning, molecular docking, and simulation, enhances vaccine design and development and shortens the required time for vaccine validation pipelines for human and veterinary uses. The next-generation sequencing technology offers strong support to the screening of some antiviral therapies and vaccine design and development through decoding the genetic materials of the currently circulating strains of the viral pathogens of interest [12]. Integration of the NGS and AI-driven tools will enhance vaccine design and development, increase the efficacy of the designed vaccines, and minimize the number of animals used for the vaccine pipelines [13,14,15]. Hence, the proposed work provides a pathway and alternative in silico vaccine design strategy that complements the existing vaccination approaches rather than replacing them. The proposed pan-parvovirus vaccine is designed to solve this problem by including conserved regions among many parvovirus strains from canine and feline species to provide wider protection against the existing and new variants.

In the current study, we intend to develop a pan multiepitope-based vaccine to possess a dual purpose for protecting both canine and feline species from dreadful parvovirus. We incorporated different computational AI tools to predict the top-ranked epitopes and validated them by analyzing immunogenic characteristic features of predicted epitopes and binding affinity scoring from docking studies with feline alleles. Immune receptors, such as TLRs, were docked with the vaccine construct, and its stability was evaluated. The vaccine construct was cloned through in silico cloning to understand its behavior in the biological living environment. This achievement has laid a solid foundation for the development of highly effective and safe multi-epitope vaccines, and meanwhile, it has provided an important preliminary basis for the prevention and control of viral infectious diseases.

## 2. Materials and Methods

### 2.1. Retrieval of the Canine and Feline Parvovirus Protein Sequences from the NCBI Database

A total of 143 isolate sequences belonging to the two structural and two non-structural proteins of canine and feline species from different regions such as China, USA, Brazil, South Korea, UK, and Australia were retrieved from the National Center for Biotechnology (NCBI) database (https://www.ncbi.nlm.nih.gov/protein, accessed on 23 July 2026). The Supplementary Table S1 presents information about these sequences.

### 2.2. The Multiple Sequence Alignment (MSA) Analysis

The Geneious software (https://www.geneious.com/, accessed on 23 July 2026) and the Clustal Omega server tool (https://www.ebi.ac.uk/jdispatcher/msa/clustalo, accessed on 23 July 2026) were used to perform multiple sequence alignment (MSA) for two non-structural and two structural proteins (NS1, NS2, VP1 and VP2) of feline and canine species [16]. The most conserved region between the structural proteins of both species was further considered for predicting B-cell and T-cell (MHC class I and class II molecules) epitopes. The conserved regions provided in Supplementary Table S2 refer to amino acid segments identified across major parvoviral proteins, including NS1, NS2, VP1, and VP2 of both feline and canine parvoviruses. These conserved regions were determined through multiple sequence alignment of each corresponding protein from FPV and CPV, and the regions showing the highest sequence identity (i.e., minimal amino acid substitutions and gaps across both species) were selected. The rationale was to capture conserved elements across both structural (VP1/VP2) and non-structural (NS1/NS2) proteins that may contribute to cross-protective immune recognition.

### 2.3. Identification of B-Cell Epitopes Within the Conserved Regions of the NS1, NS2, VP1, and VP2 Proteins

#### 2.3.1. Mapping of the Linear B-Cell Epitopes Across Parvovirus Protein Sequences

The linear B-cell epitopes were identified from the sequences of non-structural and structural proteins (NS1, NS2, VP1, and VP2) of both the feline and canine species. We used BCPREDS (BepiPred 2.0) (http://services.healthtech.dtu.dk/services/BepiPred-2.0/, accessed on 23 July 2026) [10] and the IEDB analysis resource server (http://tools.iedb.org/bcell/, accessed on 23 July 2026) for the prediction, and the length of targeted epitopes was kept to 20-mers. The antigenic properties of the predicted B-cell epitopes were analyzed using the VaxiJen server. The surface accessibility and flexibility of the epitopes were further analyzed using the Emini Surface Accessibility and Karplus–Schulz Flexibility prediction methods implemented in the IEDB.

#### 2.3.2. Mapping the Discontinuous/Conformational B-Cell Epitopes Across Parvovirus Protein Sequences

The identification of discontinuous/conformational B-cell epitopes was performed with the CBTOPE (https://webs.iiitd.edu.in/raghava/cbtope/, accessed on 23 July 2026) web server [11]. The threshold value was set to the default, and the study was conducted using the specific amino acid composition as an input feature for a support vector machine (SVM), with a prediction accuracy of more than 85% and an area under the curve (AUC) of 0.9. The individual residue-level discontinuous epitopes were predicted using the ElliPro server (https://tools.iedb.org/ellipro/, accessed on 23 July 2026), with the minimum score and maximum distance parameters set to 0.5 and 6 Å, respectively. The conformational B-cell epitopes reported at residue-level resolutionare based on the protein antigen’s 3D structure, solvent accessibility, and flexibility.

### 2.4. Mapping the T-Cell Epitopes Within the Non-Structural and Structural Proteins of Canine and Feline Species (NS1, NS2, VP1 and VP2)

#### 2.4.1. Identification of the MHC Class-I Epitopes (Cytotoxic T-Lymphocyte) 

The Cytotoxic T-lymphocyte epitopes were identified using the IEDB server (http://tools.iedb.org/main/tcell, accessed on 23 July 2026). The epitope-binding predictor NetMHCpan 4.1 BA (version 2023.09) was used to sort the peptides by the percentile rank score, and they were subjected to fundamental characteristic analysis to be selected as the best-ranking epitopes [17]. Percentile rank was chosen as the primary selection metric because it provides a normalized measure of binding affinity across different MHC alleles and is widely recommended for comparative epitope prioritization. The source species was set to canine, with a peptide length of ~9 to 10 mers and the associated canine alleles (DLA-8803401, DLA-8850101, and DLA-8850801). Canine alleles were used to predict epitopes in the feline species because their sequences were conserved, as confirmed by multiple sequence alignment (MSA).

#### 2.4.2. Identification of MHC Class-II Epitopes (Helper T-Lymphocyte)

The helper T-lymphocyte epitopes were identified using NetMHCIIpan-4.3 (https://services.healthtech.dtu.dk/services/NetMHCIIpan-4.3/, accessed on 23 July 2026), pan-specific binding of peptides to MHC class II molecules of known sequence using the experimentally predicted feline alleles (DRB1_0103, DRB1_0102 and DRB1_0107) in comparison with canine and human alleles [18,19]. The characteristic analysis was used, as previously described, and the filtered epitopes were used for further analysis.

### 2.5. Evaluation of the Cytokine Production Potential (IFNs and IL-4)

The immune response of HTL epitopes has been evaluated through their cytokine-inducing potential (IFNs), utilizing the IFN prediction software (https://webs.iiitd.edu.in/raghava/ifnepitope, accessed on 23 July 2026) [20]. This prediction helps us understand the intensive upregulating effect on exhibiting antigens present on both MHC class I and class II molecules. It primarily increases the expression of MHC class I molecules compared to MHC class II molecules. This was followed by an analysis using the Interleukin-4 (IL-4) prediction server (https://webs.iiitd.edu.in/raghava/il4pred, accessed on 23 July 2026) [20] to predict the capacity of the filtered epitopes to induce IL-4, which is essential for regulating immune homeostasis. In addition, this Th2-associated cytokine increases the expression of MHC class II molecules on antigen-presenting cells, such as B-cells and macrophages, promoting antigen presentation while having minimal impact on MHC class I.

### 2.6. Analysis of the Interaction Between the T-Cell Epitopes (MHC Class I and MHC Class II Molecules) with Feline Alleles (MHC I and MHC II Class) Through Molecular Docking

The PEP-FOLD3 (De Novo peptide structure prediction) server (https://bioserv.rpbs.univ-paris-diderot.fr/services/PEP-FOLD3/, accessed on 23 July 2026), using the sOPEP energy function, was used to cluster peptide conformations (3D structures). The sequence of the feline alleles (MHC class I—Uniprot ID: Q95481, MHC class II—Uniprot ID: Q19430) were reterived from Uniprot, and their structure were generated using Alphafold 2 colab (https://colab.research.google.com/github/sokrypton/ColabFold/blob/main/AlphaFold2.ipynb#scrollTo=UGUBLzB3C6WN, accessed on 23 July 2026). The molecular docking analysis was performed using HADDOCK server tool between the selected epitopes and feline alleles (MHC class I and II epitopes) [21]. The Biovia Discovery Studio was used to visualize the peptide—ligand interaction, key residues, and information about hydrogen bond acceptorsand donors. The epitopes with the best-binding score were selected to incorporate into the final vaccine construct.

### 2.7. Assembly of the Multi-Epitope Parvovirus Vaccine Using the Top-Ranked Epitopes

The construction of the final vaccine was performed by linking the B-cell and T-cell predicted epitopes that were filtered out based on the predicted (antigenic, non-allergic, non-toxic, and good solubility) properties of the selected epitopes. The N-terminal ends of the vaccine construct were linked with Salmonella enterica flagellin Flic adjuvants (GGS), along with the PADRE sequence, by EAAAK. The top-ranked B- and T-cell epitopes were linked using KK, AAY, and GPGPG as linkers [22,23,24]. The sequence was provided with a 6×His-tag (H) and stop codon (TAA) attached to the C-terminus for purification and identification of the vaccine upon expression. The predicted epitopes were mapped onto the protein structures and visualized using Biovia Discovery Studio to assess their surface exposure. Additionally, solvent-accessible surface area (SASA) analysis was performed to estimate the accessibility of epitope residues to antibody recognition (Figure 3). BLASTp-short analysis against canine and feline proteomes was performed to assess potential host cross-reactivity of the selected epitopes using the NCBI BLAST database (https://blast.ncbi.nlm.nih.gov/Blast.cgi?PAGE=Proteins, accessed on 23 July 2026).

### 2.8. Disulfide Bond Engineering Analysis of the Multi-Epitope Parvovirus Vaccine Construct

The importance of creating the disulfide bonds between the cysteine residues would be essential to keep the protein more stable. After refinement, the vaccine protein was further submitted to the Disulfide by Design v2.12 web server (http://cptweb.cpt.wayne.edu/DbD2/, accessed on 23 July 2026) for disulfide bridging [25]. The χ^3^ value and the Cα-Cβ-Sγ angle were kept as default parameters. As previous studies suggested, the χ^3^ angle must range between −87 and +97°, and the energy score must not exceed 2.2 kcal/mol for disulfide bridging. In the end, to allow bridging between possible residue pairs, they had undergone mutation to cysteine residues by applying the server’s “Create/View Mutant” option.

### 2.9. Evaluation of the Stability of the Designed Vaccine Construct Using the Normal Mode Analysis (NMA)

The stability and physical mobility of the atoms from the constructed vaccine molecules were analyzed using normal mode analysis (NMA). It was conducted in the iMODS server tools (https://imods.iqf.csic.es/, accessed on 23 July 2026), where multiple descriptors such as eigenvalues, covariance, B-factors, and deformability and intrinsic motions of a multiplex of proteins were analyzed [26]. The assessment of motif stiffness was performed using eigenvalues, whereas the deformity of the main chain was predicted from the biological targets’ efficacy measurement.

### 2.10. Assessment of the Physicochemical Properties of the Designed Multi-Epitope Parvovirus Vaccine Construct

We utilized the same methodology as described in our previous work; the physicochemical properties of the vaccine construct were assessed using the Protparam server (https://web.expasy.org/protparam/, accessed on 23 July 2026). The potent antigenicity of selected proteins was predicted by using the VaxiJen v2.0 server (http://www.ddg-pharmfac.net/vaxijen/VaxiJen/VaxiJen.html, accessed on 23 July 2026) with a default threshold of 0.4. The allergenicity and toxicity of proteins were assessed by using the AllerTOP v.2.1 server (http://ddg-pharmfac.net/AllerTOP/, accessed on 23 July 2026), and the ToxinPred2 server (https://webs.iiitd.edu.in/raghava/toxinpred2/index.html, accessed on 23 July 2026), respectively. The solubility nature of the final vaccine construct was analyzed using the innovagen solubility check server (https://www.innovagen.com/tools/, accessed on 23 July 2026).

### 2.11. Prediction of the Secondary and Tertiary Structures of the Designed Multi-Epitope Parvovirus Vaccine Construct

We utilized the PDBsum server tool (https://www.ebi.ac.uk/thornton-srv/databases/pdbsum/, accessed on 23 July 2026) to predict and visualize the secondary structure, topology, and folds of the final vaccine construct. The tertiary structure was modeled using AlphaFold 2 Colab, and the stability was analyzed through the Ramachandran plot from both Biovia Discovery Studio and the PDBSum server tool.

### 2.12. Molecular Docking of the Designed Multi-Epitope Parvovirus Vaccine Construct with the Feline and Canine Toll-like Receptors (TLRs)

The toll-like receptors, such as TLR4 (Uniprot ID: AF-P58727) and TLR5 (Uniprot ID: A0A1P8NLQ2), were selected to analyze the interaction between the final pan–feline–canine vaccine construct and immune receptors. The protein structures were retrieved from the Uniprot database, and the structures were visualized through Biovia Discovery Studio (v22.1.021297). Upon protein preparation, the active binding sites were projected before performing the docking studies, which is crucial for greater binding affinity. We utilized the Zdocker docking tool for performing docking, and the detailed analysis of the binding interfaces was characterized by the PDBSum server tool [27].

### 2.13. Molecular Dynamics Simulation of the Designed Multi-Epitope Parvovirus Vaccine Construct with the Feline and Canine Toll-like Receptors (TLRs)

To perform MD simulations, we prepared the receptor-peptide complexes using the Protein Preparation Wizard tool included in Maestro version 13.8.140 (Release 2023-4), which allowed adding the side chains in the peptides, minimizing the energies, and optimizing the bond angles and lengths. MD simulations were run using the Desmond molecular dynamics engine bundled within the Schrödinger software suite on a Linux-x86_64 platform. The system parameters were defined using the S-OPLS all-atom force field. The complexes were solvated in cubic boxes with the TIP3P water model. Na^+^ and Cl^−^ ions (specifically, 168 Na^+^ ions, 178 Cl^−^ ions, and 10 explicit Cl^−^ counterions were added to neutralize the net charge of the system, bringing the final setup to a total of 16,643 atoms. After system minimization using the steepest descent integrator, equilibration protocols were executed under NVT and NPT conditions. The relaxation outputs were subsequently used for a production MD simulation under NPT ensemble conditions at a constant temperature of 300.0 K for a total trajectory length of 10 ns. Coordinates were saved at regular intervals throughout the run. Trajectory files containing the coordinates of the macromolecular complex were used to evaluate structural stability and convergence within the binding area. The final simulation structures were visualized with Maestro by Schrödinger. Analyses of root mean square deviation (RMSD), root mean square fluctuation (RMSF), and local interaction energies were carried out for the MD simulations of each system using the trajectory analysis tools within the suite [28,29].

### 2.14. Codon Optimization and In Silico Cloning of the Multi-Epitope Parvovirus Vaccine Construct

As previously described, the multiepitope vaccine was optimized and cloned into the expression vector to ensure the potential effective cloning. Hence, the reverse translation of the vaccine protein sequences into a respective DNA sequence was performed using the VectorBuilder software (https://en.vectorbuilder.com/tool/codon-optimization.html, accessed on 23 July 2026). The codon adaptive index (CAI) value and the GC content of the multi-epitope construct were also calculated as described previously. The restriction enzyme sequences BamHI and EcoRI were added at the DNA’s 3′ and 5′ ends, respectively. Along with this, the Kozak sequence was added to ensure efficient translational initiation in eukaryotic expression systems, which surrounds the starting codon. The restriction cloning module from Snapgene V.6.0.2 software was used to incorporate the multi-epitope construct into the pET28a(+) plasmids using the indicated restriction enzyme sites. The pET-28a(+) vector was selected solely for in silico cloning to evaluate the feasibility of recombinant protein expression and molecular cloning in a bacterial expression system. Its use in this study does not imply the development of a eukaryotic DNA vaccine but rather serves as a preliminary assessment of the construct’s suitability for recombinant protein production [30,31].

### 2.15. In Silico Immune Simulation of the Designed Multi-Epitope Parvovirus Vaccine Construct

The immune response to the designed multiepitope vaccine was predicted using an in silico immune simulation on the C-ImmSim server (https://150.146.2.1/C-IMMSIM/index.php, accessed on 23 July 2026). The C-ImmSim server simulates the influence of vaccine construct on B and T lymphocytes by modeling the immune system of three major immune organs (bone marrow, thymus, and spleen). The parameters were set as default with 50 and 1000 simulation steps [32]. We are proposing the administration of the designed multiepitope vaccine construct three times using 4-week intervals. During the simulation, each step represented eight hours of real time, corresponding to time points of 1, 84, and 168 h. Subsequently, this server was also used to predict the host cellular immune response and cytokine expression levels induced by the multi-epitope vaccine candidates in silico, as previously described.

## 3. Results

### 3.1. Results of the Prediction of the B-Cell Epitopes (Linear and Discontinuous) Within the Major Proteins of Feline and Canine Parvoviruses (NS1, NS2, VP1 and VP2)

The IEDB and BCpred server tools were utilized as described in our previously published work, and the results of those two servers were kept with the default threshold value of 0.75. The length of the peptides was kept at around 20-mers. This length was chosen because linear B-cell epitopes predicted by the server naturally vary in size, and a peptide length of around 20-mer residues is commonly used in vaccine design to ensure adequate antigenic coverage and surface accessibility. The threshold value of >0.75 is used and refers to the default confidence score provided by the selected prediction server, where scores closer to 1 indicate a higher probability of being a true B-cell epitope. The analyzed epitopes (NS1-9, NS2-4, VP1-12 and VP2-9) were subjected to antigenicity prediction. Among those peptides, the top-ranked epitopes with high antigenic scores were taken into consideration for the final vaccine construct (Table 1). The Emini surface accessibility and Karplus–Schulz Flexibility prediction methods implemented in the IEDB database (B-cell prediction server) were performed to analyze the surface-exposed region of the protein recognized by antibodies, shown in Supplementary Figure S1, and the flexibility of the protein surface associated with potential antigenic epitopes, shown in Supplementary Figure S2.

The discontinuous/conformational epitope prediction was performed with cbtope server utilizing the FASTA sequence of the considered proteins with a minimum score of −0.3. The list of the recognized discontinuous B-cell epitopes at different exposed surface areas is shown in Table 2. The individual residues from the predicted discontinuous epitopes through the Ellipro database on the surface of the 3D structure of all the considered proteins were visualized, and the solvent accessibility area was highlighted and shown in Supplementary Tables S3–S6.

### 3.2. Results of the Prediction of the Cytotoxic T-Lymphocyte Epitopes (MHC Class-I Molecules) Within the Major Proteins of the Feline and Canine Parvoviruses (NS1, NS2, VP1 and VP2)

As previously stated in the materials and methods, MHC class I molecules and its associate cytokine induction (IFN) were analyzed using the IEDB and IFN server tool. The selected canine alleles were chosen based on their availability in the IEDB database, and these alleles represent only a fraction of the MHC diversity present in global canine populations. However, because experimentally validated canine MHC alleles remain limited in publicly available databases, these alleles provide the most appropriate dataset for conducting the analysis. Table 3 shows the predicted MHC class I epitopes from conserved sequences of canine and feline species, analyzed based on the percentile ranking score and the characteristic features such as allergenicity, antigenicity, non-toxicity, and solubility values. Table 4 shows the predicted IFN epitopes using the support vector machine (SVM) method; the epitopes, which show a positive value with their desired score, were considered for the final vaccine construct.

### 3.3. Prediction of Helper T-Lymphocyte Epitopes Within the Major Proteins of Feline and Canine Parvoviruses (NS1, NS2, VP1, and VP2)

The predicted MHC class II molecules and the cytokine-inducing epitopes (IL-4) were analyzed, and the values are tabulated. The use of canine MHC class II alleles for predicting feline epitopes was based on previously published literature, reporting conserved structural features and similarities in peptide-binding regions of mammalian MHC class II molecules. A reference study demonstrated comparable binding preferences and structural conservation that support limited cross-species applicability in computational epitope prediction. The predicted epitopes were filtered based on their physicochemical properties (Table 5) and subjected to cytokine induction based on the SVM method (Table 6). The values that showed a positive result were the final candidates to be used in the vaccine construct.

### 3.4. Results of Molecular Docking of Selected MHC Class I and II Epitopes with the Major Proteins of Feline and Canine Parvovirus (NS1, NS2, VP1 and VP2)

**Figure 1:**
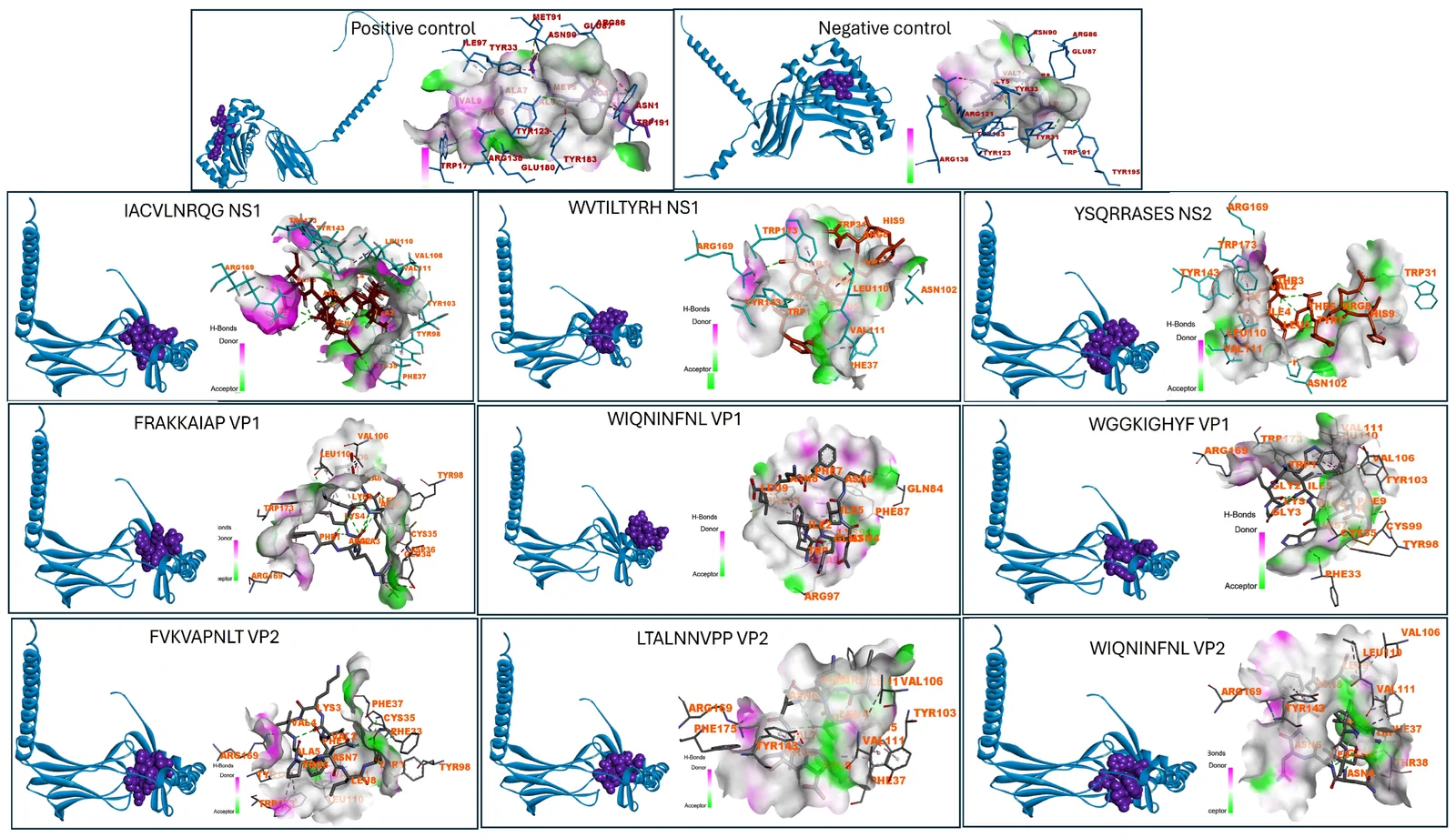
Docking analysis between MHC class I feline allele and selected epitopes: WVTILTYRH NS1, IACVLNRQG NS1, YSQRRASES NS2, FRAKKAIAP VP1, WGGKIGHYF VP1, WIQNINFNL VP1, FVKVAPNLT VP2, LTALNNVPP VP2, and WIQNINFNL VP2.

**Figure 2:**
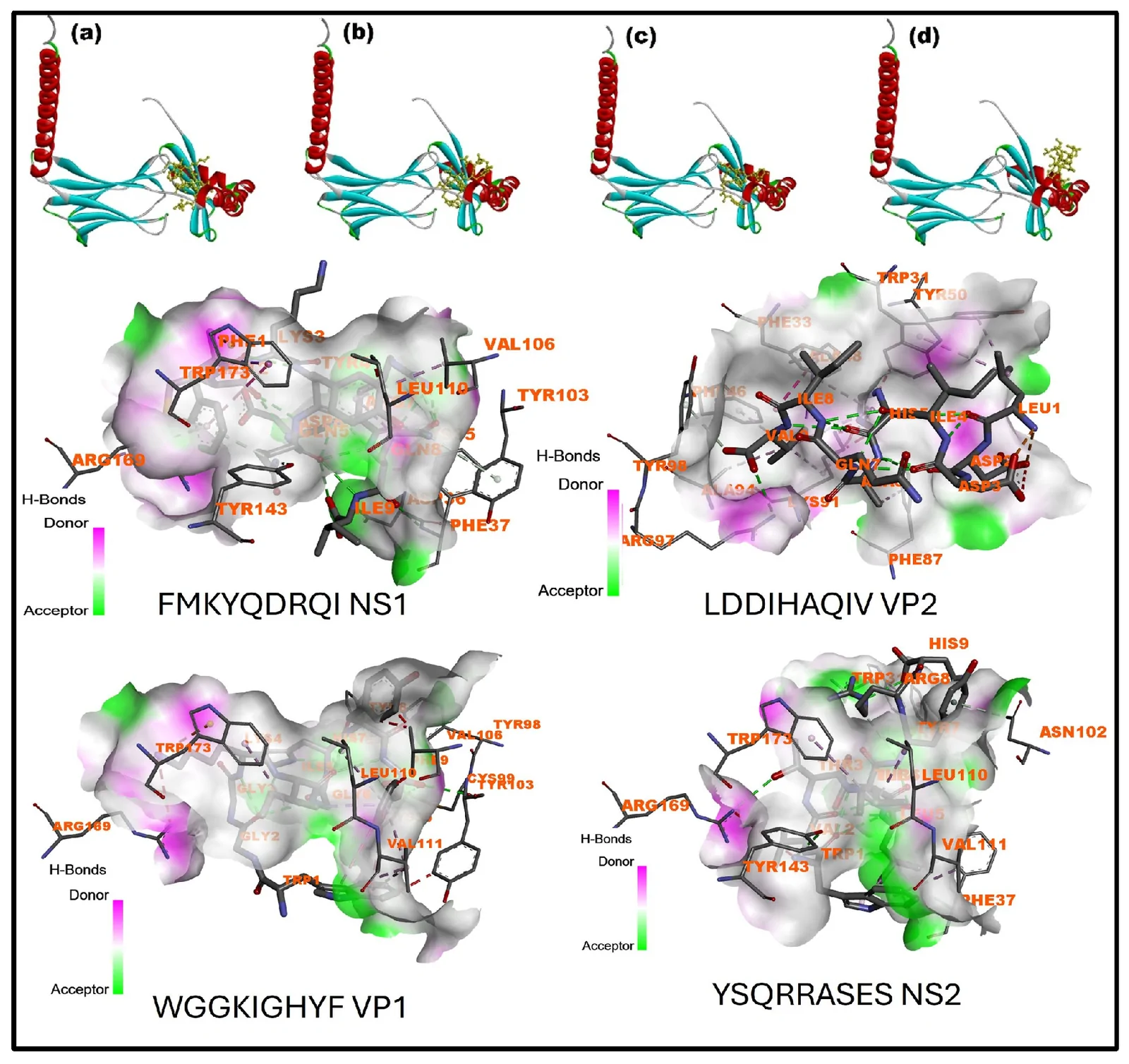
Docking analysis between MHC class II feline allele and selected epitopes: (**a**) FMKYQDRQI NS1; (**b**) YSQRRASES NS2; (**c**) WGGKIGHYF VP1; (**d**) LDDIHAQIV VP2.

**Figure 3:**
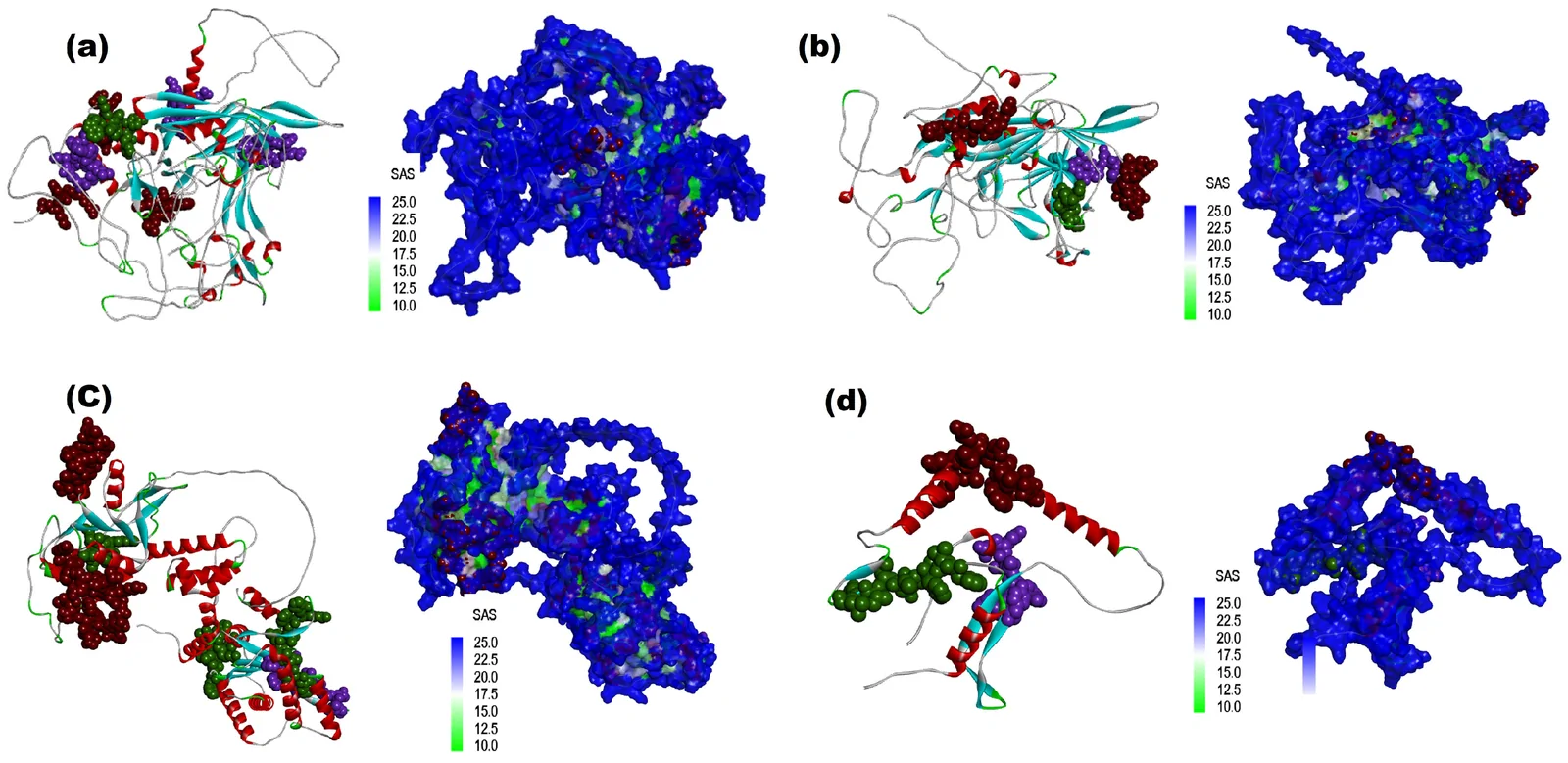
Epitope mapping and solvent accessibility analysis of the viral proteins: (**a**) NS1 showing the predicted B-cell, MHC class I, and MHC class II epitopes mapped onto the protein sequence and corresponding solvent-accessible surface; (**b**) NS2 showing the predicted B-cell, MHC class I, and MHC class II epitopes and solvent-accessible surface; (**c**) VP1 showing the predicted B-cell, MHC class I, and MHC class II epitopes and solvent-accessible surface; and (**d**) VP2 showing the predicted B-cell, MHC class I, and MHC class II epitopes and solvent-accessible surface regions.

The interactions between the Feline MHC I and MHC II antigens (DRB—(Uniprot ID: Q95481—MHC class I and Uniport ID: Q19430—MHC class II)) and the selected epitopes were studied using the HADDOCK server tool using peptide-binding groove affinity (Figure 1 and Figure 2). The figures show the interacting residues as well as the hydrogen bond donors and acceptors involved in the interactions. Initially, we utilized the PEPFOLD3 server to design the structure of epitopes/peptides and import PDB files into the HADDOCK server. The feline DRB files were used as receptors, and the MHC class I and MHC class II peptides listed in Table 3, Table 4, Table 5 and Table 6 were considered as ligands. The experimentally validated MHC class I-restricted CMV epitope NLVPMVATV was used as a positive control because of its well-established strong MHC binding properties. A low-affinity peptide, PQRLGAVSL, was used as a negative control to represent weak peptide–MHC interactions. The results show the binding affinity and confidence score, as listed in Table 7, and the top-ranked epitopes showing the highest binding affinity score were chosen for the design of the final vaccine construct. Figure 3 shows mapping of those highly ranked B-cell, MHC class I and class II epitopes with the respective protein structure (NS1, NS2, VP1 and VP2) and its Solvent accessibility surface (SAS) regions.

### 3.5. Structure and Design of the Multiepitope Vaccine Against the Major Proteins of the Feline and Canine Parvovirus (NS1, NS2, VP1 and VP2)

**Figure 4:**
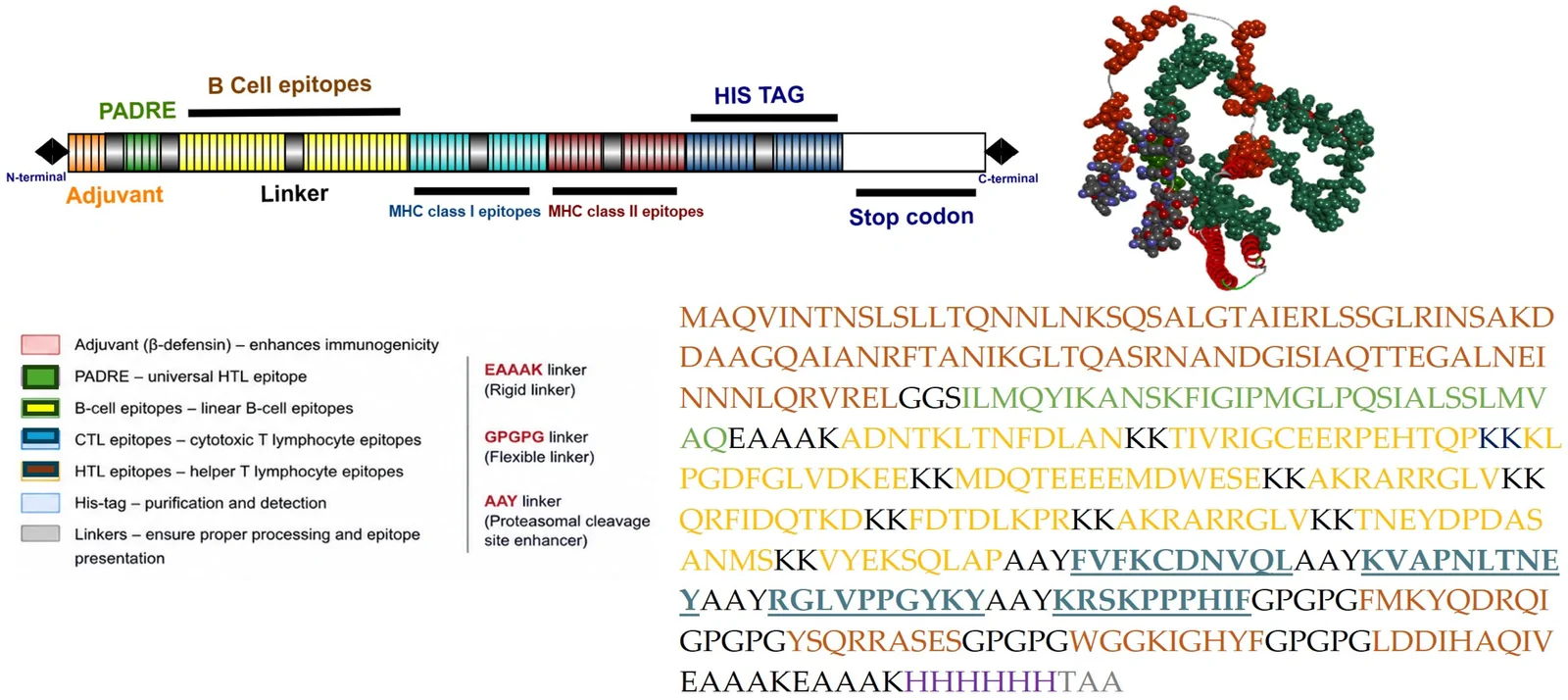
Schematic representation of the multiepitope vaccine construct targeting the top-ranked epitopes from the major structural proteins (NS1, NS2, VP1 and VP2) of feline and canine parvovirus (**top left**). Mapping of the predicted B-cell, MHC class I, and MHC class II epitopes across viral proteins (**top right side**). Red indicates B-cell epitopes, gray represents MHC class I epitopes, and green represents MHC class II epitopes. The sequence arrangement of the final multiepitope vaccine construct comprising the selected epitopes genetically linked to the adjuvants to enhance immunogenicity is shown at the bottom right.

The final vaccine was designed by combining the top-ranked B-cell epitopes and T-cell epitopes of both MHC I and MHC II classes of molecules, filtered for high antigenicity, non-allergic, non-toxic, and good solubility, and with the better binding affinity score of the structural proteins (NS1, NS2, VP1, and VP2) of feline and canine parvovirus, as listed in Table 5. The N-terminus was linked to the adjuvants and PADRE using GGS and EAAAK linkers, whereas the C-terminus was linked to a 6× His tag (HHHHHH) and a stop codon using an EAAAK linker. Linkers such as KK, AAY, and GPGPG were used to connect top-ranked B-cell and T-cell epitopes (Figure 4). BLASTp-short analysis against the canine and feline proteomes revealed that a few epitopes exhibited partial similarity to host proteins, whereas most of the epitopes showed no significant matches. The identified hits did not demonstrate complete high-identity alignment across the full epitope length, suggesting a low probability of potential autoimmune cross-reactivity (Supplementary Excel File S1).

### 3.6. Results of the Physicochemical Properties of the Designed Multiepitope Vaccine Against the Feline and Canine Parvoviruses (NS1, NS2, VP1 and VP2)

The physicochemical properties, such as molecular weight, Theoretical pI, number of amino acids, formation of Cys residues, instability index, and nature of solubility of the constructed vaccine, were analyzed using the Protparam server tool. The results showed that the vaccine had a molecular weight of 43,165.02 Da and a theoretical isoelectric point (pI) of 9.71, indicating its alkaline nature. The number of positively charged residues (Arg + Lys) was 62, the number of negatively charged residues (Arg + Glu) was 41, and the extinction coefficient measured in water at 280 nm was shown to be 0.706, indicating that the vaccine construct could be readily quantified using UV-vis spectrometry during the expression and purification procedure. The instability index (II) was about 10.30, showing that the structure of the vaccine protein was stable. The aliphatic index was about 69.19, showing moderate thermostability, and the GRAVY (Grand average of hydropathicity) value of −0.736 indicated the hydrophilic nature, showing favorable interaction with aqueous environments and potentially enhanced solubility.

### 3.7. Results of the Secondary and Tertiary Structures of the Designed Multiepitope Parvovirus Vaccine Construct

**Figure 5:**
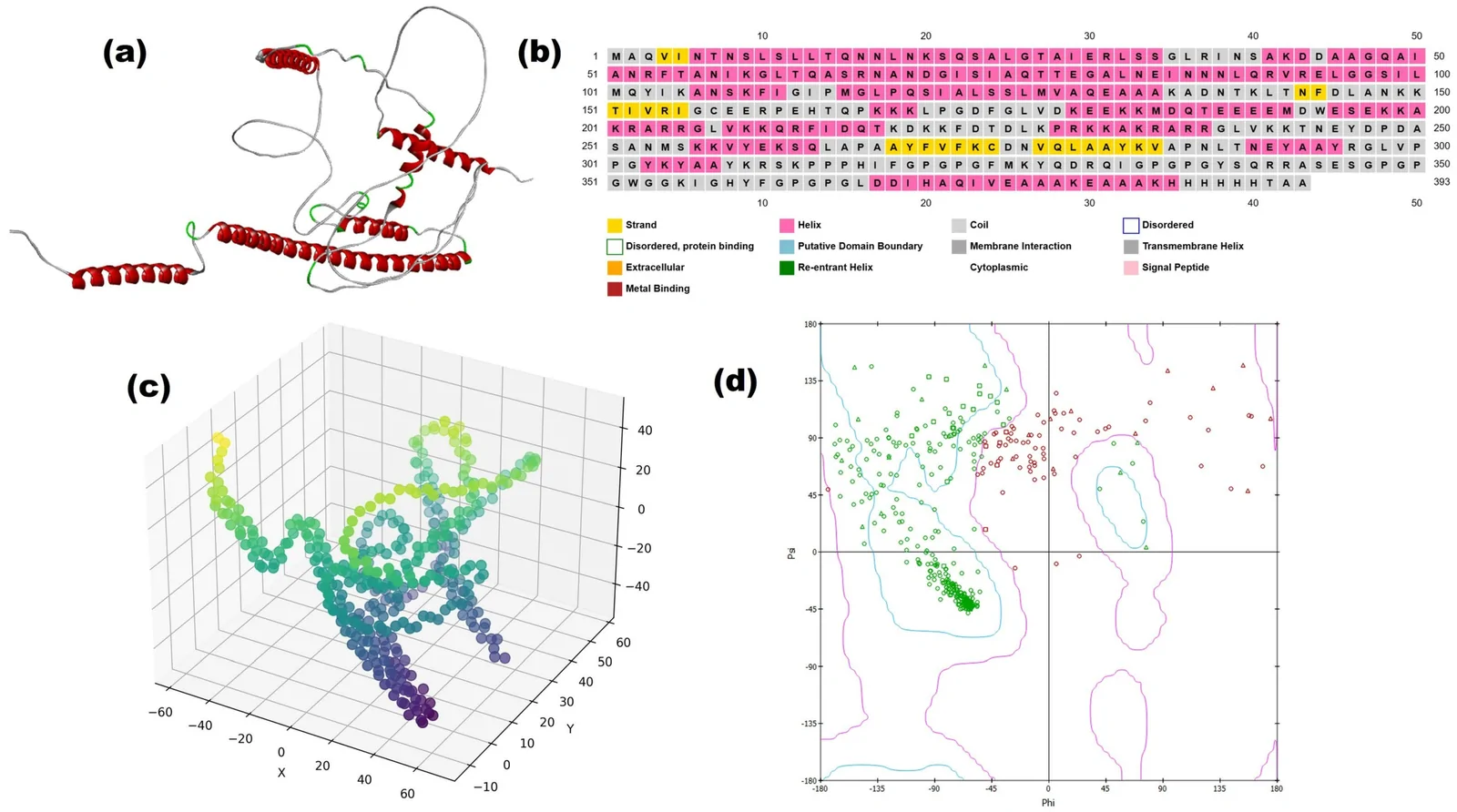
Structural analysis of the final multi-epitope vaccine construct (**a**) Three-dimensional structure of the vaccine construct modeled through Biovia Discovery Studio using the template sequence alignment method, (**b**) Topology diagram of the multi-epitope vaccine construct showing the arrangement of the secondary structure elements, (**c**) Three-dimensional representation of the spatial arrangement of the C-alpha atoms predicted using the AlphaFold prediction model, (**d**) Ramachandran plot confirming the structural stability and quality of the predicted protein conformation, with most residues located in the favored regions.

The secondary and tertiary structure of the multiepitope-based vaccine construct were analyzed and modeled through the Biovia Discovery Studio and Psipred server tool (Figure 5a,b). We utilized the AlphaFold prediction model to predict the structural features with a 3D XYZ coordinate plot (Figure 5c). The coordinate plot shows a well-organized and compact spatial distribution of C-alpha atoms, including the epitopes, linkers, and adjuvant elements, folded into a stable conformation. The stability of the constructed vaccine was analyzed by a Ramachandran plot, as previously described (Figure 5d).

### 3.8. Results of the Disulphide Bond Engineering

The analysis from the Disulfide by Designv2.12 webserver showed that the six residue pairs, such as ASN-SER, LEU-ALA, PRO-GLY, PRO-TYR, GLU-SER, and PRO-ASP, were observed based on chi3 and B-factor energy parameters.

**Figure 6:**
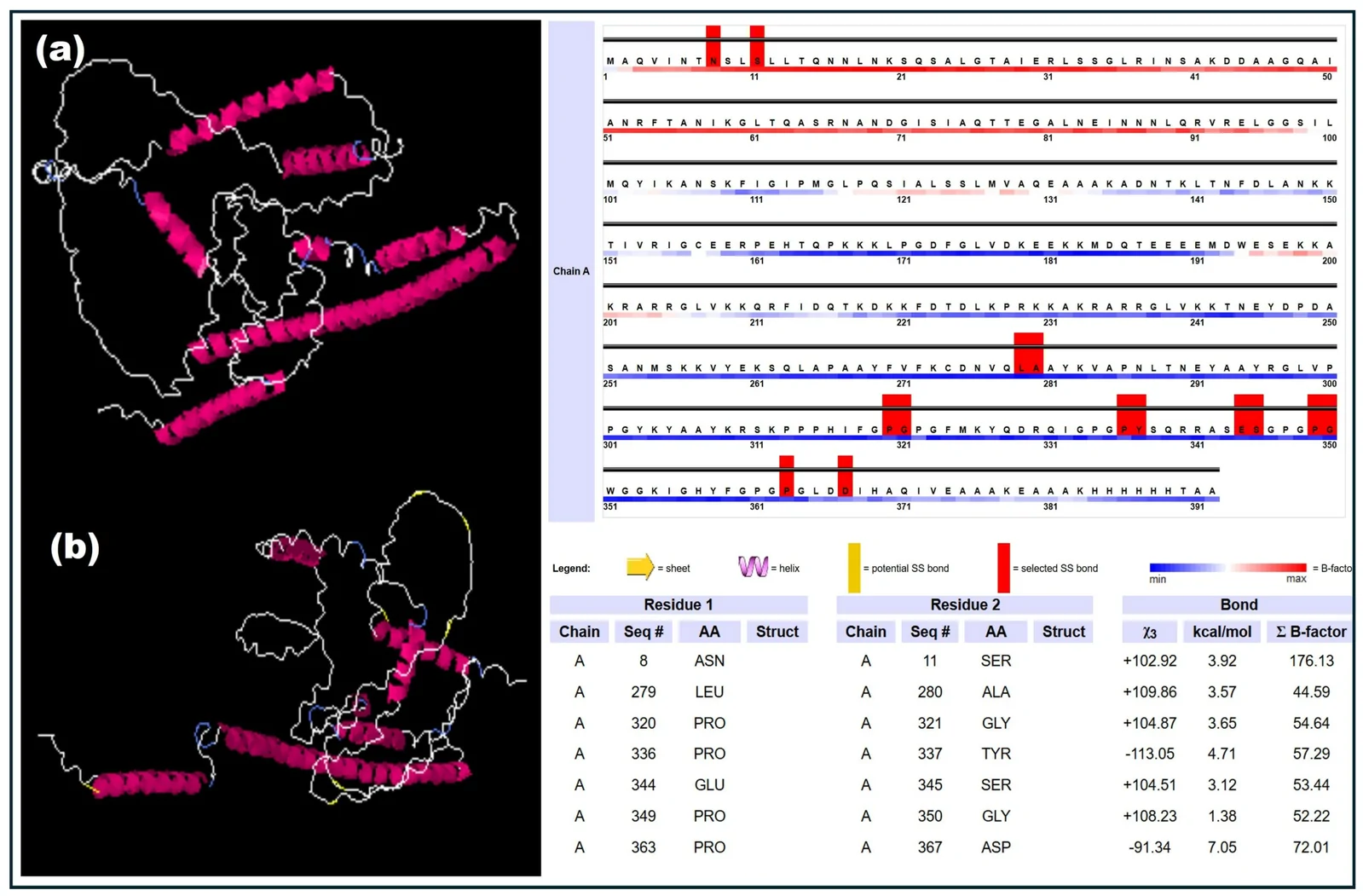
Stability of the vaccine construct following disulfide bond engineering: (**a**) original form and (**b**) mutant form. Six pairs of amino acids, highlighted with red sticks, show the S-S bonds and their corresponding X3 and energy values (left side).

In each scenario, the formation of disulfide bonds was initiated through the introduction of cysteine residues, and the evaluation was performed within the range of −87 to +97 chi3 values, ensuring an energy value of <2.2. These steps were employed to identify the suitable residues for the formation of disulfide bonds, enhancing the stability of the vaccine construct (Figure 6).

### 3.9. Results of the Assessment of the Stability of the Designed Vaccine Construct Using the Normal Mode Analysis and Prediction

**Figure 7:**
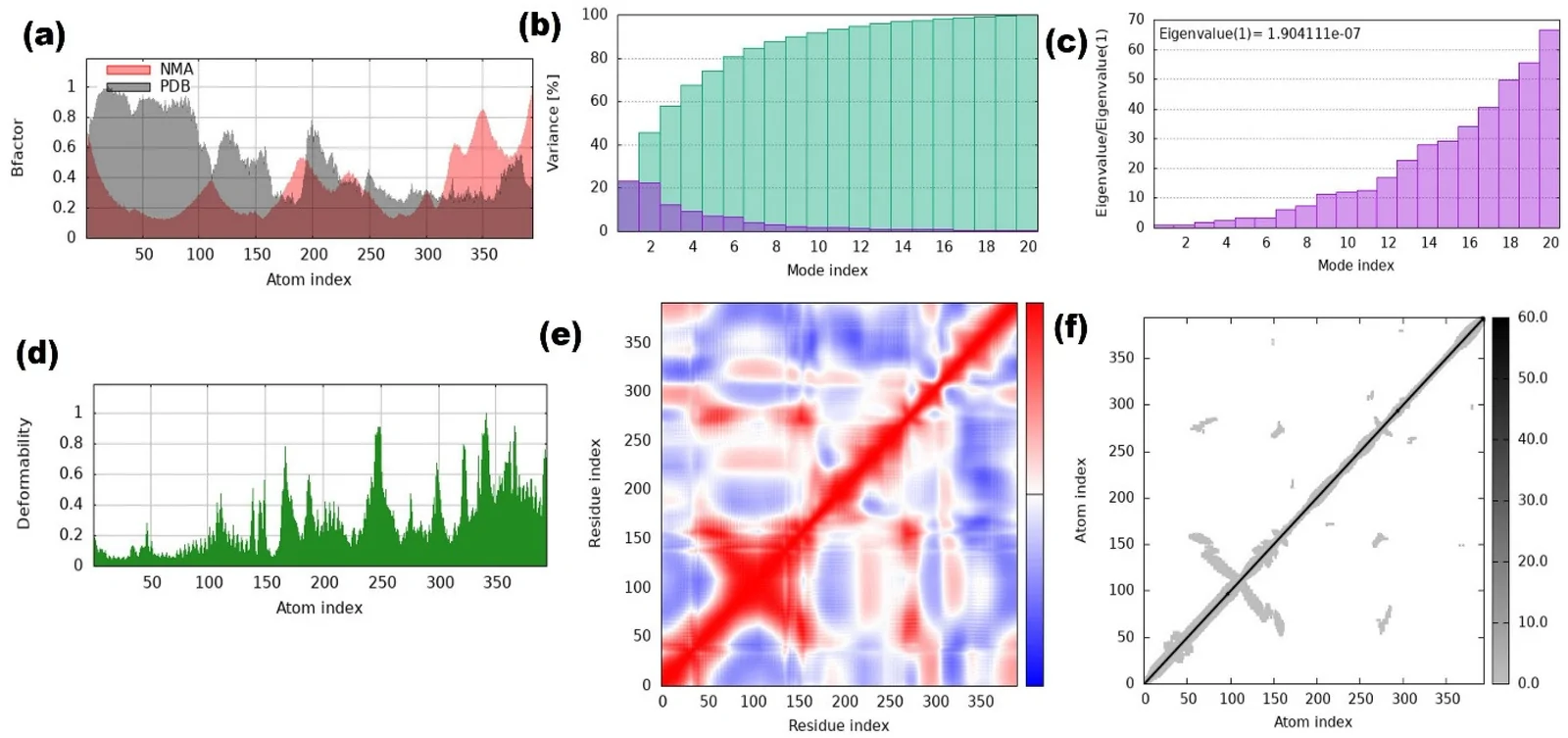
Normal-mode analysis of the vaccine protein: (**a**) B-factor—NMA mobility, (**b**) variance (purple color indicates individual variances, and green color indicates cumulative variances), (**c**) eigenvalues, (**d**) deformability, (**e**) covariance map (correlated (red), uncorrelated (white), or anti-correlated (blue) motions), and (**f**) elastic network.

The stability and physical movement of the vaccine complex were analyzed with normal mode analysis using the iMODS server tool. The figure showed that the B factor (Figure 7a) represents the atomic fluctuations in the normal mode analysis (NMA) compared with the experimental mode of vibration (PDB). Values below 50 show stable proteins, whereas values above 50 indicate flexible proteins. The variance plot showed restricted motion through purple and green colors reflecting the cumulative variance (Figure 7b). The eigenvalue of the complex showed a value of about 1.904111 × 10^−7^, as shown in Figure 7c, and the deformability plot (Figure 7d) showed the capability of bending during the motion. Figure 7e shows the covariance map illustrating the motion correlations between residues, where red indicates correlated movements, blue indicates anti-correlated movements, and white shows no movement between them. Lastly, the elastic network showed the stable region with dense springs and the flexible region with sparse ones (Figure 7f).

### 3.10. Results of the Molecular Docking of the Designed Vaccine Construct with the Feline and Canine Toll-like Receptors (TLR4 and TLR5)

**Figure 8:**
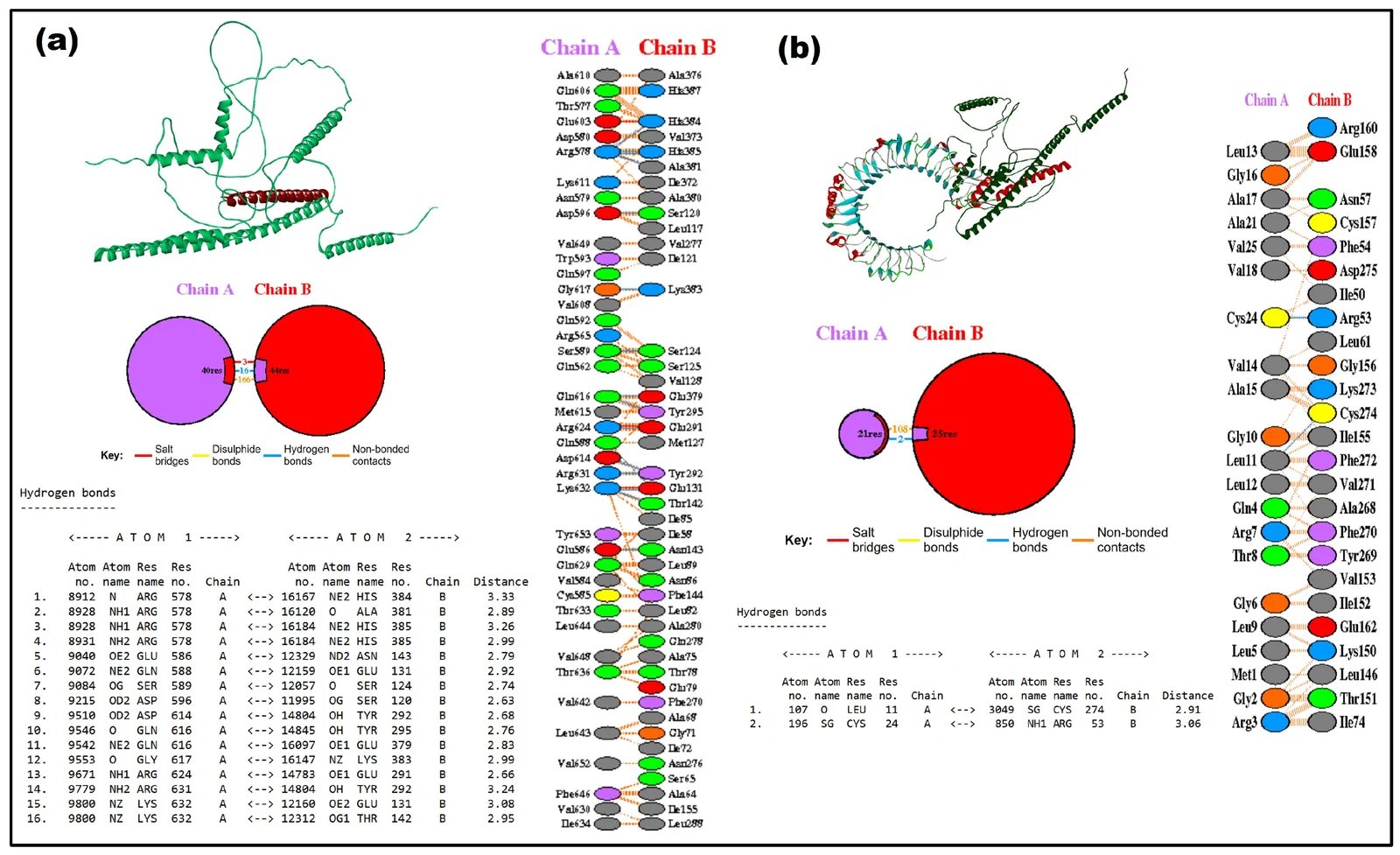
Molecular docking analysis of the multiepitope vaccine construct with the feline and canine toll-like receptors (TLR4 and TLR5) using BIOVIA Discovery Studio. (**a**) Docked complex showing the binding interaction between the vaccine construct and feline TLR4 and visualization of the interacting residues and list of binding amino acid residues involved in the interaction with feline TLR 4. (**b**) Docked complex showing the binding interaction between the vaccine construct and canine TLR5 and visualization of the interacting residues and list of binding amino acid residues involved in the interaction with canine TLR 5, as identified through PDBsum analysis.

Exploring the interactions between the vaccine construct and the toll-like receptors of both feline and canine species is important for developing a more effective pan-vaccine candidate for preventing parvovirus infection in both species. Hence, the molecular docking analysis was performed on the vaccine construct and toll-like receptors (TLR4–feline and TLR5–canine). Initially, the sequences of TLR4 (Uniprot ID: AF-P58727) and TLR5 (Uniprot ID: A0A1P8NLQ2) were retrieved from the database and modeled through Biovia Discovery Studio, followed by preparing the protein for the docking study by removing water molecules, adding hydrogen, and performing energy minimization. Docking analysis was performed using ZDock, and the results obtained indicate the strong binding affinity between the vaccine construct and toll-like receptors (TLR4 and TLR5). The top-ranked complexes, together with their respective ZDOCK scores, confirmed the strong and stable interactions between the molecules (Figure 8). The interaction residues, multiple hydrogen bonds, and hydrophobic bonds were analyzed through the PDBsum server tool. Figure 8a,b shows the topology visualization of TLR4 and TLR5, the docking interaction analysis between the vaccine construct and toll-like receptors, and the PDBsum server analysis of the interacting amino acid residues and the formation of multiple hydrogen bonds and hydrophobic interactions. Supplementary Figure S3 illustrates Solvent Accessible Surface Area (SASA) analysis of the pure and docked vaccine construct and toll-like receptors.

### 3.11. Results of the Molecular Dynamics Simulation of the Designed Vaccine Construct with Feline and Canine Toll-like Receptors (TLR4 and TLR5)

**Figure 9:**
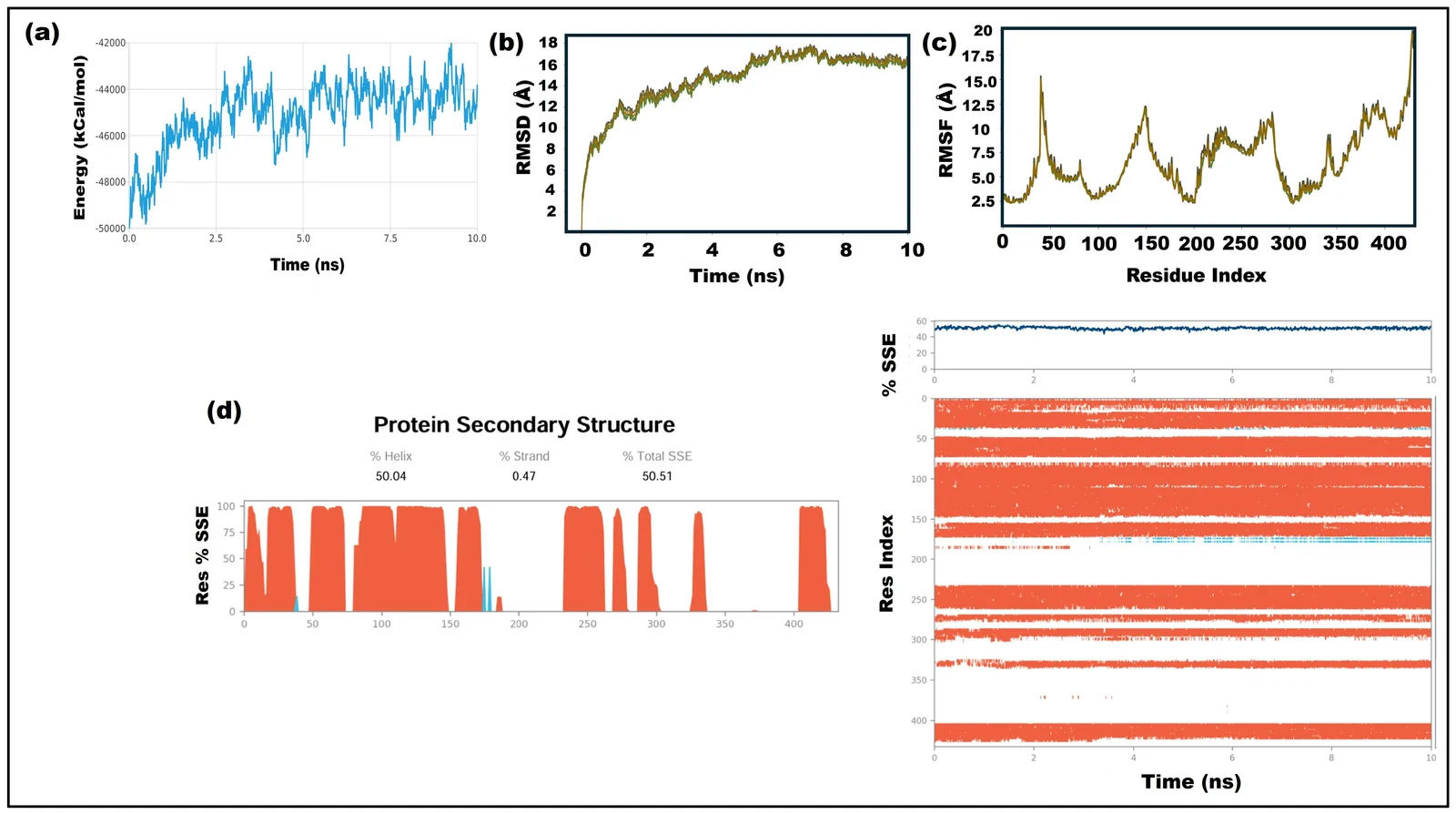
Molecular dynamic simulation analysis of the multiepitope vaccine construct with the feline toll-like receptor (TLR4) using the Desmond molecular dynamics engine bundled within the Schrödinger software suite over 10 ns, (**a**) Potential energy profile showing the stabilization of the protein structure, (**b**) Root mean square deviation (RMSD) graph showing the structural deviation of the protein structure, (**c**) Root mean square fluctuation (RMSF) graph showing the residue wise flexibility across the protein structure, (**d**) Secondary structure element (SSE) analysis providing the distribution of α-helices and β-strands.

**Figure 10:**
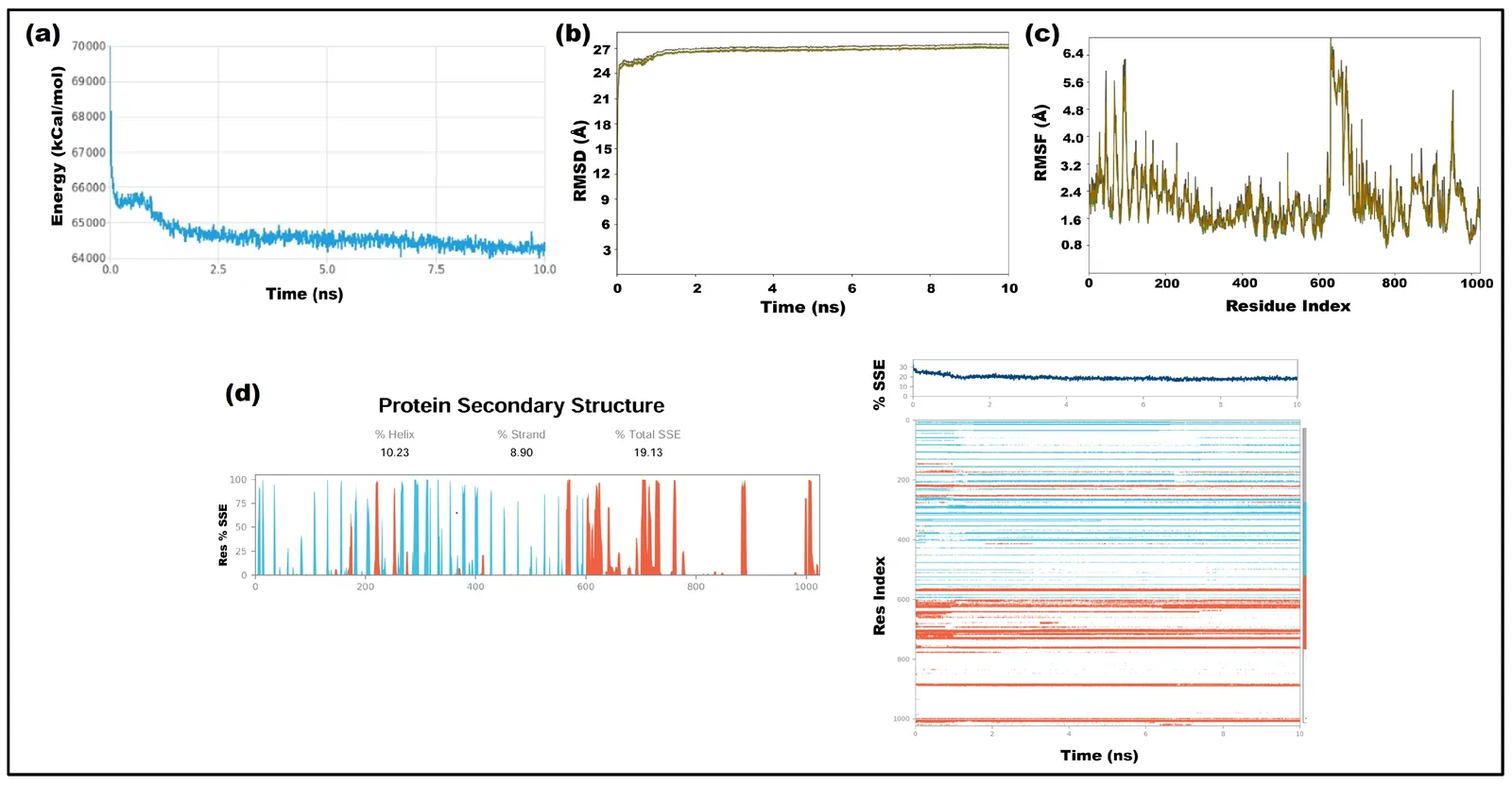
Molecular dynamic simulation analysis of the multiepitope vaccine construct with the canine toll-like receptor (TLR5) using the Desmond molecular dynamics engine bundled within the Schrödinger software suite over 10 ns, (**a**) Potential energy profile showing the stabilization of the protein structure, (**b**) Root mean square deviation (RMSD) graph showing the structural deviation of the protein structure, (**c**) Root mean square fluctuation (RMSF) graph showing the residue wise flexibility across the protein structure, (**d**) Secondary structure element (SSE) analysis providing the distribution of α-helices and β-strands.

The results showed the molecular dynamics simulation analysis performed for 10 ns between the toll-like receptors (TLR4-feline (Figure 9) and TLR5-canine (Figure 10)) and the vaccine construct. Figure 9a shows that the potential energy profile begins with moderate fluctuations and transitions to a stable mode throughout the simulation period (TLR4). Figure 9b,c displays the root mean square deviation (RMSD) and root mean square fluctuation (RMSF) graphs, showing a gradual increase in the initial stage followed by the value reaching approximately 16–17 Å with relative stability. The RMSF graph showed the residue flexibility of the protein sequence, where it resulted in higher fluctuations in some regions than in other regions, displaying moderate fluctuations. Figure 9d reveals information about the residue changes through secondary structure analysis throughout the simulation; the total secondary structural element (SSE) in the protein structure was about 50.51%, consisting of 50.04% α-helices (red color) and 0.47% β-strands (blue color), and it remained consistent throughout the 10 ns trajectory.

Figure 10 shows the molecular dynamics simulation (10 ns) of canine (TLR5) toll-like receptor with the final vaccine construct, demonstrating the gradual stabilization of the system energy. The potential energy value showed a decreased range of ~67,000 kcal/mol at 0 ns to ~64,500 kcal/mol at 10 ns, with minor fluctuations throughout the trajectory (Figure 10a). The RMSD profile indicated an early equilibration phase within the first ~0–1 ns, followed by a relatively stable trajectory within the range of 24–27 Å (Figure 10b). Figure 10c shows the RMSF graph, which provides residue-wise flexibility with fluctuations ranging from ~0.8 Å to 6.4 Å. The elevated flexible regions around specific residue clusters are above ~5 Å (Figure 10c). Figure 10d shows the secondary structure analysis, which shows a total of 19.13%, comprising 10.23% α-helix and 8.90% β-strand, and the profile remained relatively consistent throughout the 10 ns simulation, with no major structural collapse observed (Figure 10d).

### 3.12. In Silico Cloning of the Multi-Epitope Vaccine Spanning Key Epitopes from the Major Proteins (NS1, NS2, VP1 and VP2)

The vaccine construct was cloned using the Vector Builder from the decoded amino acid sequence of each epitope’s respective DNA sequences to mimic the vaccine’s expression in the *E. coli* K12 expression system. The GC content and codon adaptation index values generated by the Vector Builder server represent the level of expression in the *E. coli* system. Finally, SnapGene software was used to clone the constructed vaccines in the pET-28a(+) expression vector between the restriction enzyme cutting locations of *BamHI* and *EcoRI*, and the results obtained are shown in Supplementary Figure S4a,b.

### 3.13. In Silico Immune Simulation of the Designed Multi-Epitope Vaccine Containing Key Epitopes from the Major Proteins (NS1, NS2, VP1 and VP2)

The predicted immune response of the constructed vaccine was analyzed through the interaction between the antigens and B-cells, T-cells, and cytokines (Supplementary Figure S5a–s) utilizing C-ImmSim immune simulation methods. The primary and secondary humoral responses were shown by a significant increase in IgM and IgG. Similar to our IFN-γ and IL-4 database prediction, the marked expansion and robust cellular immune response were evaluated with this immune simulation as well.

## 4. Discussion

Canine and feline parvoviruses have become among the most dreaded viral infections affecting dogs and cats and are characterized by marked leukopenia, high fever, and hemorrhagic diarrhea, often resulting in severe dehydration, secondary infections, and mortality rates exceeding 80% in susceptible animals [1]. Dogs and cats under two years of age with acute bloody diarrhea should be considered at high risk for parvovirus infection, particularly if their vaccine history is incomplete. Since both feline panleukopenia virus (FPV) and its close corollary canine parvovirus (CPV) are antigenically stable, i.e., they will not change rapidly as other viruses do, the existing vaccines remain very effective. But the animals in shelters across the country are still losing their lives daily to these diseases [33,34]. Hence, there is an urgent need to develop some effective vaccines that could protect both species against those dreadful viruses. As described in our previously published articles, the incorporation of AI into the vaccine development process has vastly improved. The multi-epitope vaccine not only provides long-term protection by inducing trained immunity but is also safer and more cost-effective. However, its major limitation is the need for extensive validation before clinical application [35,36].

The project mainly focuses on utilizing AI-integrated tools to develop a multiepitope-based vaccine against both feline and canine parvovirus. Initially, it begins with (i) retrieving the desired sequence of feline and canine structural and non-structural proteins (NS1, NS2, VP1, and VP2) from the NCBI database and performing MSA analysis for the conserved regions. (ii) The B-cell and MHC class I epitopes were predicted from the IEDB database, and MHC class II epitopes were predicted with NetMHCIIpan-4.3,using the experimentally predicted feline alleles (DRB1_0103, DRB1_0102, and DRB1_0107) in comparison with canine and human alleles (percentile score (<4) for MHC class I molecules and (<10) for MHC class II molecules. This was followed by (iii) an analysis of characteristic features, including antigenic, non-allergic, non-toxic, and better solubility (VaxiJen 2.0 and Aller Top). (iv) The top-ranking epitopes were docked with feline MHC class I and II alleles to analyze the binding interaction between them (HADDOCK docking server). (v) The final vaccine was constructed with top-ranked epitopes through linkers and adjuvants, and the physicochemical properties of the designed vaccine were studied. (vi) Molecular docking and molecular dynamics simulation were performed between the vaccine construct and TLRs (TLR4 and TLR5) of feline and canine toll-like receptors to study the binding interaction between them (Biovia Discovery Studio and Schrödinger software suite). (vii) Immunosimulation of the final vaccine construct was performed to assess its potential potency in the activation of the humoral and cell-mediated immunity of both feline and canine species (Immsim).

The major challenge in this study was the prediction of MHC class II epitopes, as there is a lack of data on feline alleles in online database servers. This has been rectified through alternative strategies by using the experimentally predicted alleles aligned with human and canine alleles in identifying the epitopes with the NetpanMHCII class server tool [37,38,39]. The parameters were default, with the polymerase length of 12 mer. This approach successfully provided the best score data and matched the feline alleles with the corresponding epitopes.

The interferons (IFNs) and Interleukins (IL-4) play the most important part in triggering the immune response in both MHC class I and MHC class II molecules of T-cell expression. The filtered epitopes of MHC class I molecules were subjected to the interferon SVM method of prediction, and the epitopes, such as FVFKCDNVQL (0.45722211), KVAPNLTNEY (0.43379691), RGLVPPGYKY (0.45733135), and KRSKPPPHIF (0.44701719), showed positive values. The filtered epitopes of MHC class II molecules were subjected to the interleukin SVM method of prediction, and the epitopes, such as FMKYQDRQI (1.24), IACVLNRQG (0.29), WVTILTYRH (0.28), YSQRRASES (0.30), WGGKIGHYF (0.28), and LDDIHAQIV (0.24), resulted in positive values. The top-ranked peptide was selected based on its high antigenic score; non-allergic, non-toxic, and solubility scores from two structural and two non-structural genomes were subjected to molecular docking studies using the HADDOCK server and confirmed their binding interaction with Feline MHC class I and MHC class II molecules. The list of residual interactions along with the hydrogen bond information was visualized through the Biovia Discovery Studio.

The binding score values for MHC class I and MHC class II molecules were provided as follows: MHC class I of molecules—FVFKCDNVQL (0.7526 and −193.10 docking score—NS1), KVAPNLTNEY (0.8912 and −157.61 docking score—NS2), RGLVPPGYKY (0.5816 and −214.32 docking score—VP1), KRSKPPPHIF (1.1015 and −201.88 docking score—VP2) and MHC class II of molecules—FMKYQDRQI (0.8792 and docking score of −190.38—NS1), YSQRRASES—(0.5799 and docking score of −227.71—NS2), WGGKIGHYF—(1.0920 and docking score of −222.16—VP1) and LDDIHAQIV—(0.9059 and docking score of −168.88—VP2). Finally, these epitopes were used in the vaccine design and were constructed using linkers and adjuvants. The solvent accessible surface area (SASA) analysis provided the surface exposure of pure and docked complexes. The buried surface area values were calculated for the vaccine construct (45,550.4 Å^2^), TLR4 (46,737.2 Å^2^), and TLR5 (4244.46 Å^2^) in their unbound forms, as well as for the complexes of the vaccine construct with TLR5 (46,797.2 Å^2^) and TLR4 (69,886.3 Å^2^). These changes in the value indicate that the receptor binding altered the solvent accessible surface of the interacting proteins, showing stable complex formation. Higher SASA values with TLR4 show greater exposure and better interaction than TLR5, ensuring stronger binding affinity and structural integrity. Thus, the analysis suggests that the vaccine construct retains its structural stability while changing its structure to adapt for better binding (Supplementary Figure S3). Like previous studies, molecular docking with toll-like receptors TLR4 and TLR5 has been widely used to predict potential vaccine–receptor interactions, though experimental validation is required to confirm receptor binding and downstream immune activation [39,40].

The disulfide bond analysis was performed to predict the presence of cysteine residues, which contain a reactive thiol (-SH) group in their side chain, and once incorporated into a protein, this thiol would participate in important biochemical functions. Normally, disulfide bonds form due to the oxidation reaction that happens between two cysteine residues, which makes the vaccine structure more stable. The conformational classification χ_3_ dihedral angle (C–S–S–C) describes the rotation around the S-S bond and determines the spatial orientations of the two cysteines linked by it [40]. When the angle is approximately ±90°, the bonds show the right- or left-handed spiral conformations, and when it is close to 180°, they correspond to the trans conformation, showing the extended and linear arrangement of cysteines. Finally, an angle close to 0° indicates the cis conformation, which is energetically unstable because of steric clashes and unfavorable geometry. Since all the resultant values were nearly 102° to 113°, the protein structures were making the right- or left-hand conformations with their corresponding energy values.

Next, further validation of the stability and flexibility of the vaccine construct was performed with normal mode analysis (NMA). The mobility plot shows that residues with higher peaks (0.5–2 arbitrary units) correspond to flexible loops or linker regions, whereas lower values (0–0.3 arbitrary units) indicate stable structural regions. The regions that bend during the motion were shown in the deformability plot falls within the range of 0.05–0.15. Next, atomic fluctuations were assessed using the B-factor plot, where values below 50 indicate stable proteins, whereas values above 50 indicate flexible proteins. Normally, the eigenvalues falling within 1 × 10^−6^ to 1 × 10^−4^ represent the stiffness of the protein, since the obtained values were higher than the standard range, which means the vaccine construct has greater structural rigidity and requires higher energy needed for deformation. The individual (purple) and cumulative (green) variances were observed through a variance plot, where lower individual variance values (<0.05) represent restricted motion of each mode and the accumulation of modes was represented with a rise in the cumulative curve. Finally, the elastic network model gives the stable region with dense springs and flexible areas with sparse ones.

The molecular docking analysis was performed with Biovia Discovery Studio using ZDock; it showed better binding interactions between the designed vaccine epitopes and the TLRs (TLR4–feline and TLR5–canine), facilitating effective immune recognition and the initiation of a robust immune response. The binding affinity was calculated based on ZDock score and ZRank score for both feline and canine, and they are 22.26 (ZDock score), −47.409 (ZRank score) for feline and 16.54 (ZDock score), −134.295 (ZRank score) for canine. The PDBsum results were analyzed, and the interactions between the residues that possess hydrogen bonds (Arg 578–His 384), (Gly 617–Lys 383), (Asp 596–Ser 120), (Ser 589–Ser 124), (Lys 632–Glu 131) for feline TLR4 and (Cys 24–Arg 53) for canine TLR5 with the vaccine construct. The interaction residues between them were identified through Tamarind bio (PDBsum) and were displayed in the figure, resulting in multiple hydrogen bonds and hydrophobic bonds, especially to capture their better binding interactions.

Additionally, the preliminary assessment of the structural stability and dynamic behavior of the interaction between the vaccine construct and toll-like receptors (TLR4 and TLR5) was analyzed with molecular dynamics simulation (10 ns) using Schoedinger software suite. The main perspective of this analysis was to measure the overall stability and structural fluctuations under simulated physiological conditions. The extended simulation times and additional replicates to evaluate the long-term conformational stability will be our future studies. In both complexes, the system attained a stable energetic state and stable RMSD plateau after initial equilibration, indicating conformational stabilization. The root mean square fluctuation (RMSF) analysis showed greater flexibility mainly in the loop and terminal regions of the TLR4 complex, while most residues remained relatively stable. In contrast, the TLR5 complex exhibited localized flexibility in specific regions, with the majority of residues remaining stable. The secondary structure elemental analysis (SSE) showed that throughout the 10 ns simulation period, 50.5% of the protein’s secondary structural elements were α-helices for TLR4, whereas 10.23% were α-helices and 8.90% were β-strands, suggesting preservation of the protein’s structural architecture for TLR5 with the vaccine construct [40,41].

Next, the vaccine construct was cloned using the optimized codons with vector builders to express in the *E. coli* K12 system by converting the amino acid sequence into a DNA sequence. Through SnapGene, the insertion of the vaccine was performed into the pET-28a(+) vector between BamHI and EcoRI sites, confirming proper insertion and orientation of the gene; this results in the vaccine construct being able to be efficiently expressed in *E. coli* for the production of recombinant protein [42]. Finally, in silico immune simulations using C-ImmSim provided critical insights about the potential immune responses elicited by the designed two structural and two non-structural proteins (NS1, NS2, VP1 and VP2) of the pan multi-epitope-based (canine and feline) vaccine constructs [43,44,45,46,47]. The simulation results revealed robust activation of T-cell populations, including Cytotoxic T-cells and helper T-cells, crucial for cellular and humoral immunity. As described in our previously published article, this analysis would tell us that the vaccine construct in this study would induce strong humoral and cell-mediated immunity [48].

From these results, we would like to convey that our constructed multi-epitope-based vaccine incorporating the conserved regions of two structural and two non-structural proteins (NS1, NS2, VP1 and VP2) will be effective in the protection of both canine and feline species against the parvovirus. Though computational analysis will support us in predicting the more precise top-ranking epitopes and their immunogenic properties, further experimental validation is required to be used against those viruses.

## 5. Conclusions

We successfully designed a multi-epitope-based vaccine compiling the top-ranked immunogenic and antigenic epitopes. The interferon and interleukin will enhance/trigger the immune response to those MHC class I and MHC class II molecules. The T-cell epitopes showed high binding affinities with the feline alleles. The disulfide bond prediction and normal mode analysis showed that our vaccine construct is stable and flexible. The molecular docking showed higher binding affinities and stable conformations with canine (TLR5) and feline (TLR4) toll-like receptors. Molecular dynamics simulation for 10 ns showed the extent of stability of the systems under the considered physiological conditions, and further extended simulations will be performed in future studies to investigate long-term stability and additional conformational dynamics. The designed vaccine construct showed high immunogenic potential in terms of the production of humoral and cell-mediated immunity in felines and canines using an immune simulation approach. We believe the designed vaccine in the current study will protect both the feline and canine species against this dreadful parvovirus.

## Figures and Tables

**Table 1:** Predicted antigenic linear B-cell epitopes across the conserved region of major proteins of feline and canine parvoviruses (NS1, NS2, VP1 and VP2).

No	Start	End	Peptide (IEDB)	Length	Antigen Score
**NS1**					
1	349	361	ADNTKLTNFDLAN	13	1.3722
2	491	506	TIVRIGCEERPEHTQP	16	0.8492
3	519	531	KLPGDFGLVDKEE	13	1.3432
**NS2**					
11	67	80	MDQTEEEEMDWESE	14	1.1828
**VP1**					
12	5	13	AKRARRGLV	9	1.0456
13	65	73	QRFIDQTKD	9	1.0456
15	228	241	NNMDKTAVNGNMAL	14	0.6846
16	298	309	ESATQPPTKVYN	12	0.4705
18	602	606	NVPPV	5	0.5012
19	617	624	FDTDLKPR	8	1.5012
20	650	662	TNEYDPDASANMS	13	0.7027
21	683	689	RASHTWN	7	0.6420
22	696	703	INVDNQFN	8	1.0518
23	710	724	GGMKIVYEKSQLAPR	15	0.7348
**VP2**					
24	5	13	AKRARRGLV	9	1.0456
26	188	199	FNNQTEFKFLEN	12	0.6846
30	644	656	TNEYDPDASANMS	13	0.7027
31	677	698	RASHTWNPIQQMSINVDNQFNY	22	0.6793
32	707	715	VYEKSQLAP	9	1.1655

Note: Probable antigen—shows the predicted epitopes possess antigenic properties.

**Table 2:** Predicted antigenic discontinuous/conformational B-cell epitopes across the conserved region of major proteins of feline and canine parvoviruses (NS1, NS2, VP1 and VP2).

S.No	Gene Type	Discontinuous/Conformational B-Cell Epitopes
1	NS1	MSGNQYTEEV MEGVNWLKKH AENEAFSFVF KCDNVQLNGK DVRWNNYTKP IQNEELTSLI RGAQTAMDQT EEEEMDWESE VDSLAKKQVQ TFDALIKKCL FEVFVSKNIE PNECVWFIQH EWGKDQGWHC HVLLHSKNLQ QATGKWLRRQ MNMYWSRWLV TLCSVNLTPT EKIKLREIAE DSEWVTILTY RHKQTKKDYV KMVHFGNMIA YYFLTKKKIV HMTKESGYFL STDSGWKFNF MKYQDRQIVS TLYTEQMKPE TVETTVTTAQ ETKRGRIQTK KEVSIKCTLR DLVSKRVTSP EDWMMLQPDS YIEMMAQPGG ENLLKNTLEI CTLTLARTKT AFELILEKAD NTKLTNFDLA NSRTCQIFRM HGWNWIKVCH AIACVLNRQG GKRNTVLFHG PASTGKSIIA QAIAQAVGNV GCYNAANVNF PFNDCTNKNL IWIEEAGNFG QQVNQFKAIC SGQTIRIDQK GKGSKQIEPT PVIMTTNENI TIVRIGCEER PEHTQPIRDR MLNIKLVCKL PGDFGLVDKE EWPLICAWLV KHGYESTMAN YTHHWGKVPE WDEWAEPKI QEGINSPGCK DLETQAASNP QSQDQVLTPL TPDVVDLALE PWSTPDTPIA ETANQQSNQL GVTHKDVQAS PTWSEIEADL RAIFTSEQLE EDFRDDLD
2	NS2	MSGNQYTEEV MEGVNWLKKH AENEAFSFVF KCDNVQLNGK DVRWNNYTKP IQNEELTSLI RGAQTAMDQT EEEEMDWESE VDSLAKKLQR LRDTSGKQSS ESRPSSNSSD SGRSGPCTGT VEYSRYAYCR NCKSTIKPTW RYSQRRASES DMVRNRGRPE SHLYF
3	VP1	MAPPAKRARR GLVPPGYKYL GPGNSLDQGE PTNPSDAAAK EHDEAYAAYL RSGKNPYLYF SPADQRFIDQ TKDAKDWGGK IGHYFFRAKK AIAPVLTDTP DHPSTSRPTK PTKRSKPPPH IFINLAKKKK AGAGQVKRDN LAPMSDGAVQ PDGGQPAVRN ERATGSGNGS GGGGGGGSGG VGISTGTFNN QTEFKFLENG WVEITANSSR LVHLNMPESE NYRVVVNNMD KTVNGNMALD DIHQIVTPWS LVDANAWGVW FNPGDWQLIV NTMSELHLVS FEQEIFNVVL KTVSESATQP PTKVYNNDLT ASLMVALDSN NTMPFTPAAM RSETLGFYPW KPTIPTPWRY YFQWDRTLIP SHTGTSGTPT NYHGTDPDDV QFYTIENSVP VHLLRTGDEF ATGTFFFDCK PCRLTHTWQT NRALGLPPFL NSLPQSEGAT NFGDIGVQQD KRRGVTQMGN TYITEATIMR PAEVGYSAPY YSFEASTQGP FKTPIAAGRG GAQTDENQAA DGPRYAFGRQ HGQKTTTTGE TPERFTYIAH QDTGRYPEGD WIQNINFNLP VTNDNVLLPT DPIGGKTGIN YTNIFNTYGP LTALNNVPPV YPNGQIWDKE FDTDLKPRLH VNAPFVCQNN CPGQLFVKVA PNLTNEYDPD ASANMSRIVT YSDFWWKGKL VFKAKLRASH TWNPIQQMSI NVDNQFNYVP NIGMKIVYEK SQLAPRKLY
4	VP2	MSDGAVQPDG GQPAVRNERA TGSGNGSGGG GGGGSGGVGI STGTFNNQTE FKFLENGWVE ITANSSRLVH LNMPESENYR RVVVNNLDKT AVKGNMALDD IHAQIVTPWS LVDANAWGVW FNPGDWQLIV NTMSELHLVS FEQEIFNVVL KTVSESATQP PTKVYNNDLT ASLMVALDSN NTMPFTPAAM RSETLGFYPW KPTIPTPWRY YFQWDRTLIP SHTGTSGTPT NIYHGTDPDD VQFYTIENSV PVHLLRTGDE FATGTFFFDC KPCRLTHTWQ TNRALGLPPF LNSLPQAEGG TNFGYIGVQQ DKRRGVTQMG NTNYITEATI MRPAEVGYSA PYYSFEASTQ GPFKTPIAAG RGGAQTDENQ AADGDPRYAF GRQHGQKTTT TGETPERFTY IAHQDTGRYP EGDWIQNINF NLPVTNDNVL LPTDPIGGKA GINYTNIFNT YGPLTALNNV PPVYPNGQIW DKEFDTDLKP RLHVNAPFVC QNNCPGQLFV KVAPNLTNEY DPDASANMSR IVTYSDFWWK GKLVFKAKLR ASHTWNPIQQ MSINVDNQFN YLPNNIGAMK IVYEKSQLAP RKLY

**Table 3:** Predicted MHC class-I epitopes across the conserved region of major proteins of feline and canine parvoviruses (NS1, NS2, VP1 and VP2) and their relevant information (percentile ranks and allele specification).

Allele	Start	End	Peptide	Antigenic Score	Solubility
**NS1**					
DLA-8850101	28	37	FVFKCDNVQL	0.7526	0.4731
**NS2**					
DLA-8850101	28	37	FVFKCDNVQL	0.7526	0.4731
**VP1**					
DLA-8850801	638	647	KVAPNLTNEY	0.8912	0.5638
DLA-8803401	10	19	RGLVPPGYKY	0.5816	0.5708
DLA-8803401	113	122	KRSKPPPHIF	1.1015	0.5288
**VP2**					
DLA-8850801	638	647	KVAPNLTNEY	0.8912	0.5638
DLA-8803401	10	19	RGLVPPGYKY	0.5816	0.5708
DLA-8803401	113	122	KRSKPPPHIF	1.1015	0.5288

Note: Non-antigen, non-allergen, non-toxin—shows the predicted epitopes possess antigenic, non-allergen, and non-toxin properties.

**Table 4:** Prediction of the ability of the designed multiepitope parvovirus vaccine to trigger IFN—interferon gamma production.

Type	Allele	Method of Prediction	Inducer	Score
NS1/NS2	FVFKCDNVQL	SVM-based	POSITIVE	0.45722211
VP1/VP2	KVAPNLTNEY	SVM-based	POSITIVE	0.43379691
	RGLVPPGYKY	SVM-based	POSITIVE	0.45733135
	KRSKPPPHIF	SVM-based	POSITIVE	0.44701719

**Table 5:** Predicted MHC class-II epitopes across the conserved region of major proteins of feline and canine parvovirus (NS1, NS2, VP1 and VP2) and their relevant information (percentile ranks and allele specifications).

Pos	MHC Class II	Core	%Rank EL	Antigenic Score	Solubility
**NS1**					
237	DRB1_0103	FMKYQDRQI	6.79	0.8792	0.524
380	DRB1_0104	IACVLNRQG	9.66	1.0045	0.4634
181	DRB1_0103	WVTILTYRH	9.36	0.8681	0.4719
308	DRB1_0102	YIEMMAQPG	0.31	0.6113	0.5619
**NS2**					
139	DRB1_0103	YSQRRASES	3.14	0.5799	0.528
**VP1**					
488	DRB1_0103	FKTPIAAGR	9.35	0.4738	0.6051
83	DRB1_0103	FRAKKAIAP	1.85	1.0426	0.7233
80	DRB1_0103	HYFFRAKKA	2.91	0.5722	0.5058
703	DRB1_0102	IVYEKSQLA	0	0.7683	0.4781
588	DRB1_0102	LTALNNVPP	6.16	0.9624	0.5226
155	DRB1_0102	VRNERATGS	1.88	0.7287	0.5693
74	DRB1_0103	WGGKIGHYF	4.25	1.0920	0.4548
548	DRB1_0103	WIQNINFNL	4.54	1.1052	0.4649
643	DRB1_0107	YDPDASANM	8.66	0.5605	0.6031
633	DRB1_0102	FVKVAPNLT	2.19	0.9309	0.5289
**VP2**					
350	DRB1_0103	FKTPIAAGR	9.35	0.4738	0.6051
556	DRB1_0102	FNYLPNNIG	0.63	0.9149	0.4604
496	DRB1_0102	FVKVAPNLT	2.19	0.9309	0.5289
568	DRB1_0102	IVYEKSQLA	0	0.7683	0.4781
94	DRB1_0102	LDDIHAQIV	3.4	0.9059	0.4939
451	DRB1_0102	LTALNNVPP	6.16	0.9624	0.5226
411	DRB1_0103	WIQNINFNL	4.54	1.1052	0.4649
276	DRB1_0102	WQTNRALGL	7.5	0.6719	0.5207
506	DRB1_0107	YDPDASANM	8.66	0.5605	0.6031
448	DRB1_0102	YGPLTALNN	7.91	0.4988	0.4919

Note: Non-antigen, non-allergen, non-toxin—shows the predicted epitopes possess antigenic, non-allergen, and non-toxin properties.

**Table 6:** Prediction of IL-4-inducing epitopes.

Type	Allele	Method of Prediction	Inducer	Score
NS1	FMKYQDRQI	SVM-based	POSITIVE	1.24
	IACVLNRQG	SVM-based	POSITIVE	0.29
	WVTILTYRH	SVM-based	POSITIVE	0.28
NS2	YSQRRASES	SVM-based	POSITIVE	0.30
VP1	WGGKIGHYF	SVM-based	POSITIVE	0.28
VP2	LDDIHAQIV	SVM-based	POSITIVE	0.24

**Table 7:** List of top-ranked selected epitopes used for the construction of the multi-epitope vaccine against major proteins of the feline and canine parvovirus (NS1, NS2, VP1 and VP2).

Epitope	Types	Antigenicity	Allergenicity	Toxicity	Conservancy	Docking	IFN-γ	IL-4	Confidence Score
FVFKCDNVQL	MHC class I	0.7526	Non-allergen	Non-toxic	99.30%	−193.10	0.45722211		50.95
KVAPNLTNEY	MHC class I	0.8912	Non-allergen	Non-toxic	99.30%	−157.61	0.43379691		46.53
RGLVPPGYKY	MHC class I	0.5816	Non-allergen	Non-toxic	87.40%	−214.32	0.45733135		50.43
KRSKPPPHIF	MHC class I	1.1015	Non-allergen	Non-toxic	79.02%	−201.88	0.44701719		54.58
FMKYQDRQI	MHC class II	0.8792	Non-allergen	Non-toxic	65.03%	−190.38		1.24	46.98
YSQRRASES	MHC class II	0.5799	Non-allergen	Non-toxic	86.01%	−227.71		0.30	51.50
WGGKIGHYF	MHC class II	1.0920	Non-allergen	Non-toxic	92.30%	−222.16		0.28	45.80
LDDIHAQIV	MHC class II	0.9059	Non-allergen	Non-toxic	64.34%	−168.88		0.24	51.17
ADNTKLTNFDLAN	B-cell	1.3722	Non-allergen	Non-toxic	86.70%				
TIVRIGCEERPEHTQP	B-cell	0.8492	Non-allergen	Non-toxic	99.30%				
KLPGDFGLVDKEE	B-cell	1.3432	Non-allergen	Non-toxic	99.30%				
MDQTEEEEMDWESE	B-cell	1.1828	Non-allergen	Non-toxic	99.30%				
AKRARRGLV	B-cell	1.0456	Non-allergen	Non-toxic	87.40%				
QRFIDQTKD	B-cell	1.0456	Non-allergen	Non-toxic	92.30%				
FDTDLKPR	B-cell	1.5012	Non-allergen	Non-toxic	99.30%				
AKRARRGLV	B-cell	1.0456	Non-allergen	Non-toxic	87.40%				
TNEYDPDASANMS	B-cell	0.7027	Non-allergen	Non-toxic	99.30%				
VYEKSQLAP	B-cell	1.1655	Non-allergen	Non-toxic	99.30%				

## Data Availability

The raw data supporting the conclusions of this article will be made available by the authors upon request.

## Supplementary Materials

The following supporting information can be downloaded at: https://www.mdpi.com/article/10.3390/microorganisms14081721/s1, Figure S1: Supplementary Figure S1 shows the analysis identifies surface-exposed residues across all four proteins through Emini surface accessibility prediction of VP1, VP2, NS1, and NS2 proteins with scores above 1.0 indicating potential antibody-accessible regions in connection with B-cell epitope prediction, Figure S2: Supplementary Figure S2 shows the analysis identifies residues wise flexibility scores across all four proteins through Karplus & Schulz flexibility prediction of VP1, VP2, NS1, and NS2 proteins, where higher peaks indicate more flexible regions that may contribute to antigenic epitope formation in connection with B-cell epitope prediction, Figure S3: Supplementary Figure S3 shows the Solvent accessible surface area (SASA) analysis of Pure form of Vaccine construct, TLR4, TLR5, Vaccine construct-TLR4 complex and Vaccine construct-TLR5, where blue color indicates the exposed area and green color indicate the buried surface area, Figure S4: The vector map shows in silico cloning of the multiepitope feline and canine (NS1, NS2, VP1 and VP2) vaccine construct into the pET-28 (+) Expression Vector, Figure S5: (a–s) In silico immune simulation analysis of the multiepitope vaccine construct, The kinetics profile displaying antigen-immunoglobulin production (IgM, IgG1, IgG2, and IgM + IgG), B lymphocytes population per entity-state (i.e., showing counts for active, presenting on class-II, internalized the Ag, duplicating and anergic, B lymphocytes total count, memory cells, and sub-divided in isotypes IgM, IgG1 and IgG2, CD4 T-helper lymphocytes count sub-divided per entity-state (i.e., active, resting, anergic and duplicating), CD4 T-helper lymphocytes count. The plot shows total and memory counts, as well as CD4 T-regulatory lymphocyte count. Both total memory and per-entity-state counts are plotted: The CD8 T-cytotoxic lymphocytes count per entity-state, and the CD8 T-cytotoxic lymphocytes count. Total and memory shown, Dendritic cells. The DC can present antigenic peptides on both MHC class-I and class-II molecules, epithelial cells. The total count is broken down into active, virus-infected, and presented on class-I MHC molecule, Macrophages. Total count, internalized, presenting on MHC class-II, active and resting macrophages, Natural Killer cells (total count), Plasma B lymphocyte count subdivided per isotype (IgM, IgG1, and IgG2). The simulation was performed utilizing the antigen-combined sequence data from four proteins (NS1, NS2, VP1 and VP2) of the feline and canine parvovirus, and it was set to 100 with a volume of 10; Table S1: List of feline and canine parvovirus genome sequences retrieved from the NCBI GenBank database and used in this study. The table includes the GenBank accession number, virus/isolate name, country of origin, and collection date for each sequence. Table S2: Supplementary Table S2 shows the VP1, VP2, NS1 and NS2 gene sequences and their corresponding conserved regions identified through multiple sequence alignment. The conserved regions represent highly preserved amino acid sequences across the analyzed strains and were selected for subsequent epitope prediction and vaccine design analyses, Table S3: Supplementary Table S3 shows the Predicted conformational (discontinuous) B-cell epitopes of the VP1 protein identified using the ElliPro server. Predicted epitopes were mapped onto the three-dimensional structure of VP1 based on structural protrusion analysis. Higher protrusion index (PI) values indicate greater surface exposure and antigenic potential, suggesting an increased likelihood of antibody recognition. Table S4: Supplementary Table S4 shows the Predicted conformational (discontinuous) B-cell epitopes of the VP2 protein identified using the ElliPro server and predicted epitopes were mapped onto the three-dimensional structure of VP2 based on structural protrusion analysis, Table S5: Supplementary Table S5 shows the Predicted Conformational (discontinuous) B-cell epitopes of the NS1 protein identified using the ElliPro server and predicted epitopes were mapped onto the three-dimensional structure of NS1 based on structural protrusion analysis, Table S6: Supplementary Table S6 shows the Predicted conformational (discontinuous) B-cell epitopes of the NS2 protein identified using the ElliPro server and predicted epitopes were mapped onto the three-dimensional structure of NS2 based on structural protrusion analysis. Supplementary Excel File S1—Blastp analysis.
