# Supplementary material for: Immunoinformatics-Guided Computational Design and In Silico Validation of Multi-Epitope Vaccine Candidates Targeting Canine and Feline Parvoviruses
Source: Microorganisms. 2026 Aug 5;14(8):1721. doi: 10.3390/microorganisms14081721 (PMC13515720; doi:10.3390/microorganisms14081721)
Supplement: Supplementary file 1 [file microorganisms-14-01721-s001.zip › Supplementary Figure.pdf]

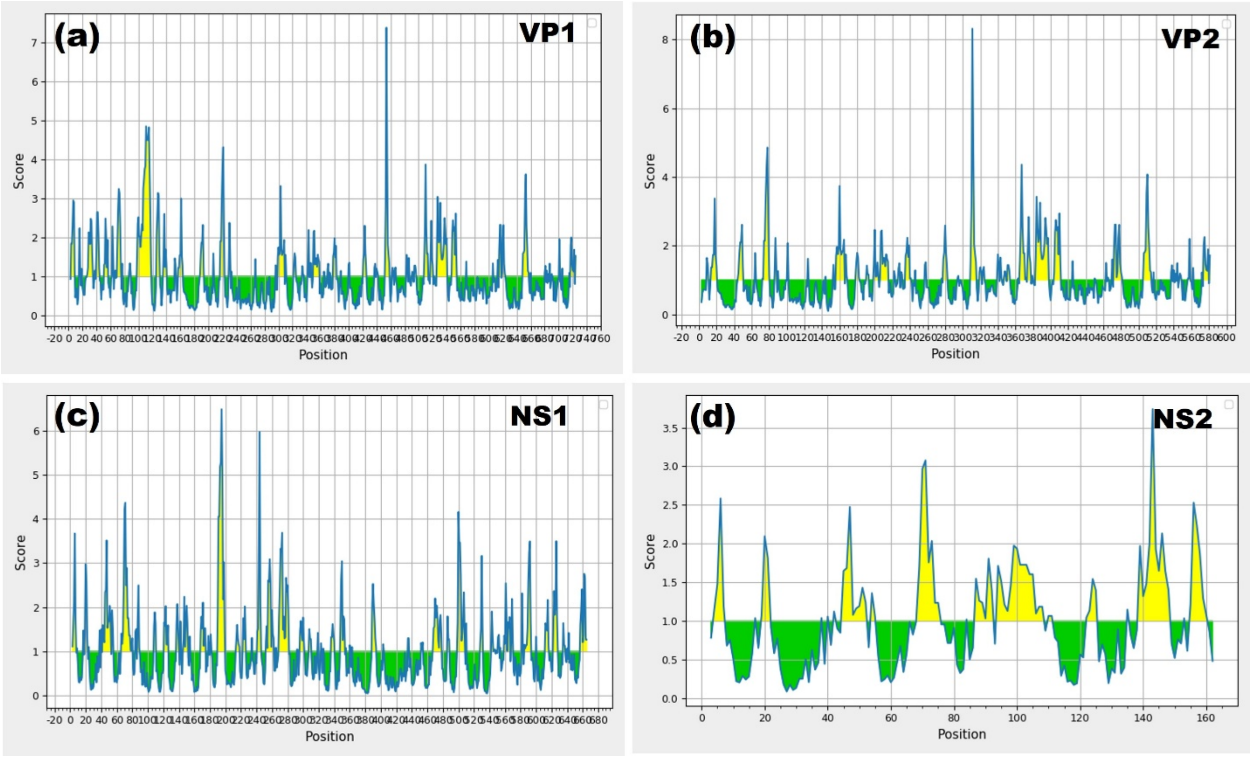

Supplementary Figure S1 shows the analysis identifies surface-exposed residues across all four proteins through Emini surface accessibility prediction of VP1, VP2, NS1, and NS2 proteins with scores above 1.0 indicating potential antibody-accessible regions in connection with B-cell epitope prediction

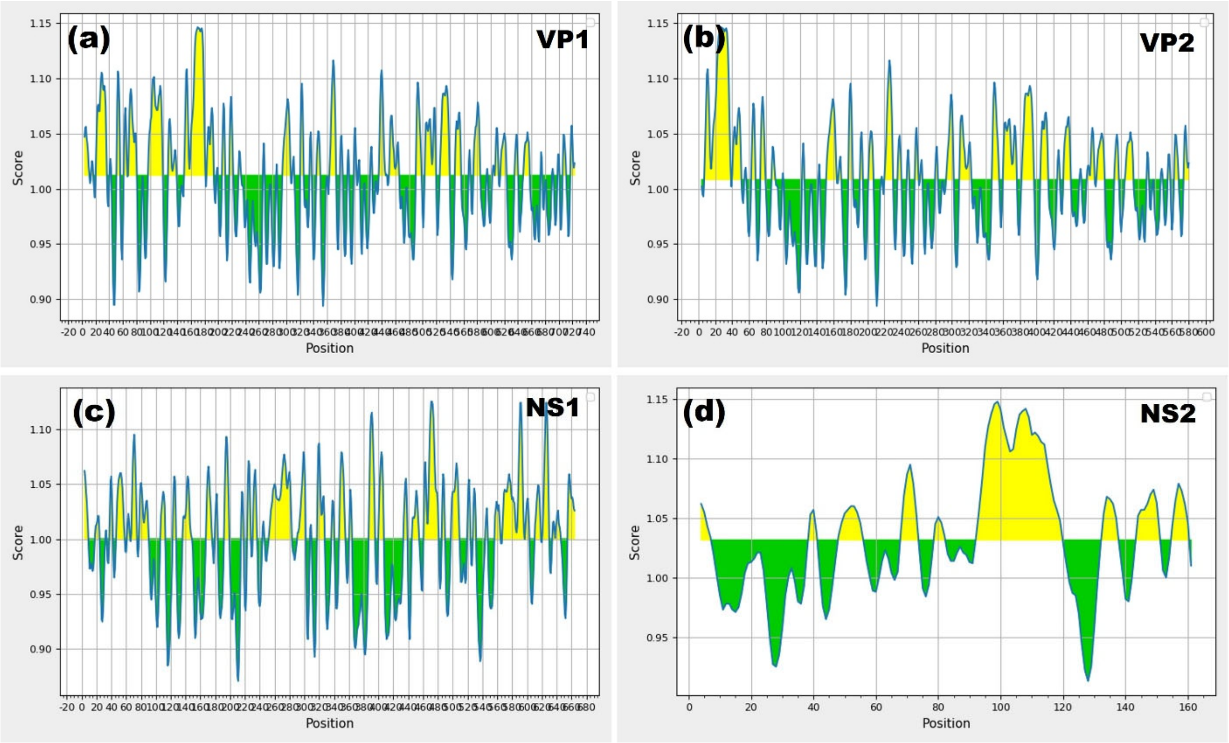

Supplementary Figure S2 shows the analysis identifies residues wise flexibility scores across all four proteins through Karplus & Schulz flexibility prediction of VP1, VP2, NS1, and NS2 proteins, where higher peaks indicate more flexible regions that may contribute to antigenic epitope formation in connection with B-cell epitope prediction

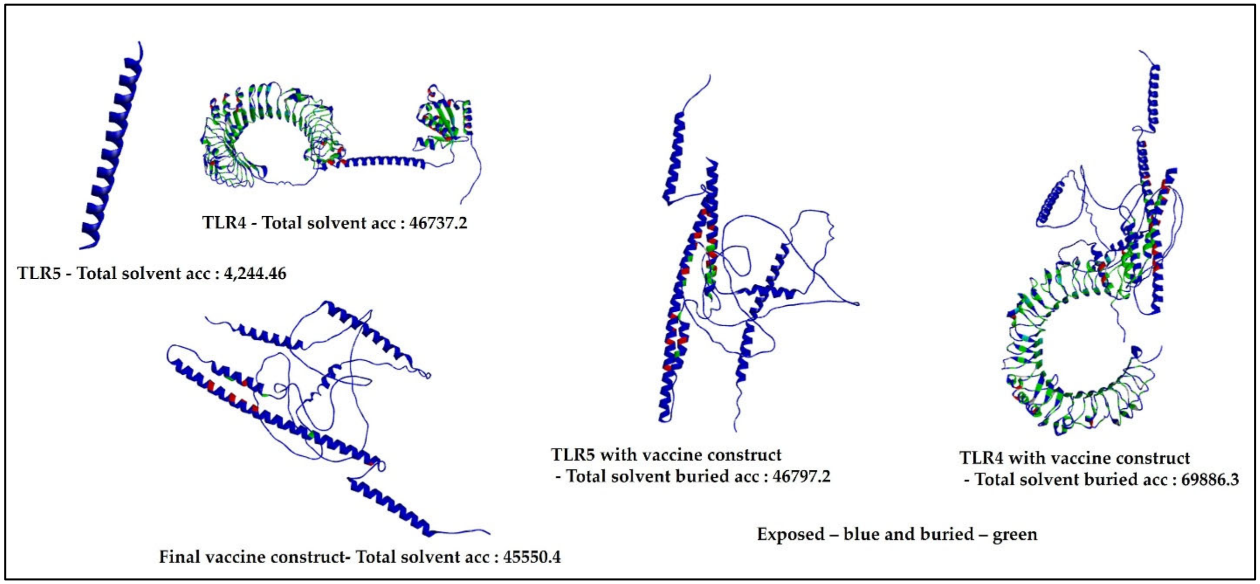

Supplementary Figure S3 shows the Solvent accessible surface area (SASA) analysis of Pure form of Vaccine construct, TLR4, TLR5, Vaccine construct-TLR4 complex and Vaccine construct-TLR5, where blue color indicates the exposed area and green color indicate the buried surface area.

Vaccine construct cloning

ATGGCGCAGGTAATTAACACCAATAGCCTGAGCCTGCTGACCCAGAATAATCTGAATAAAAGTCAGAGTGCGCTGGGCACCGCGATTGAACGCCTGAGCAGCGGCCTGCGCATTAATAG
CGCGAAAGATGATGCCGCGGGCCAGGCAATTGCGAATCGCTTTACCGCCAACATTAAAGGCCTGACCCAGGCCAGCCGCAACGCGAACGATGGCATTAGCATTGCGCAGACCAC
CGAAGGCGCGCTGAATGAAATTAATAACAACCTGCAGCGCGTGCGTGAACTGGGCGGCAGCATTCTGATGCAGTATATTAAAGCCAACAGCAAATTCATTGGCATTCCGATGGGC
CTGCCGCAGAGCATTGCCCTGAGCAGCCTGATGGTGGCGCAGGAAGCGGCGGCGAAAGCGGATAACACCAAACTGACCAACTTCGATCTGGCAAACAAAAAAACCATTGTGCG
TATTGGCTGTGAAGAACGTCCGGAACATACCCAGCCGAAAAAAAAACTGCCGGGCGATTTCGGCCTGGTGGATAAAGAAGAAAAAAAAATGGATCAGACCGAGGAAGAAGAA
ATGGATTGGGAAAGCGAAAAAAAAGCGAAACGTGCCCGTCGCGGCCTGGTTAAAAAACAGCGCTTTATTGACCAGACCAAAGATAAAAAATTTGATACCGATCTGAAACCGCGC
AAAAAGGCGAAACGTGCGCGTCGTGGCCTGGTGAAAAAAACCAATGAATATGATCCGGATGCCAGCGCCAATATGAGCAAAAAAGTGTATGAAAAAAGCCAGCTGGCGCCGGC
CGCGTATTTCGTGTTTAAATGCGATAATGTGCAGCTGGCGGCGTATAAAGTGGCGCCGAACCTGACCAACGAATATGCGGCGTATCGTGGCCTGGTGCCGCCGGGTTATAAATATGC
CGCGTATAAACGCAGCAAACCGCCTCCGCACATCTTCGGCCCGGGTCCGGGCTTTATGAAATACCAGGATCGCCAGATCGGTCCGGGCCCGTATAGCCAGCGCCGTGCGAGCGAA
AGCGGCCCGGGCCCGGGCTGGGGCGGCAAAATTGGCCATTACTTTGGCCCGGGCCCGGGTCTGGATGATATTCATGCGCAGATTGTCGAAGCCGCGGCGAAAGAAGCAGCGGCC
AAACACCATCACCATCATCATACGGCCTAA

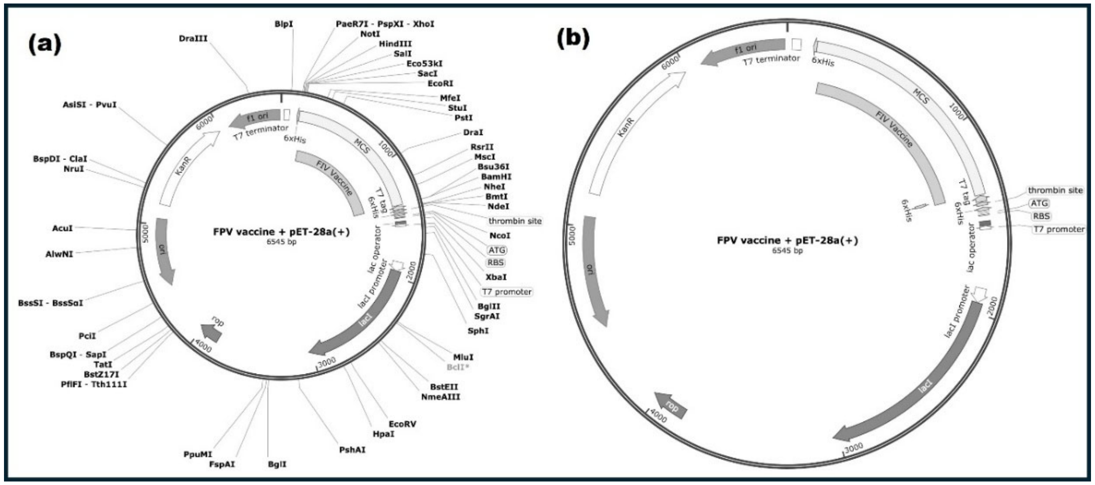

Supplementary Figure S4. The vector map shows in silico cloning of the multiepitope feline and canine (NS1, NS2, VP1 and VP2) vaccine construct into the pET-28 (+) Expression Vector

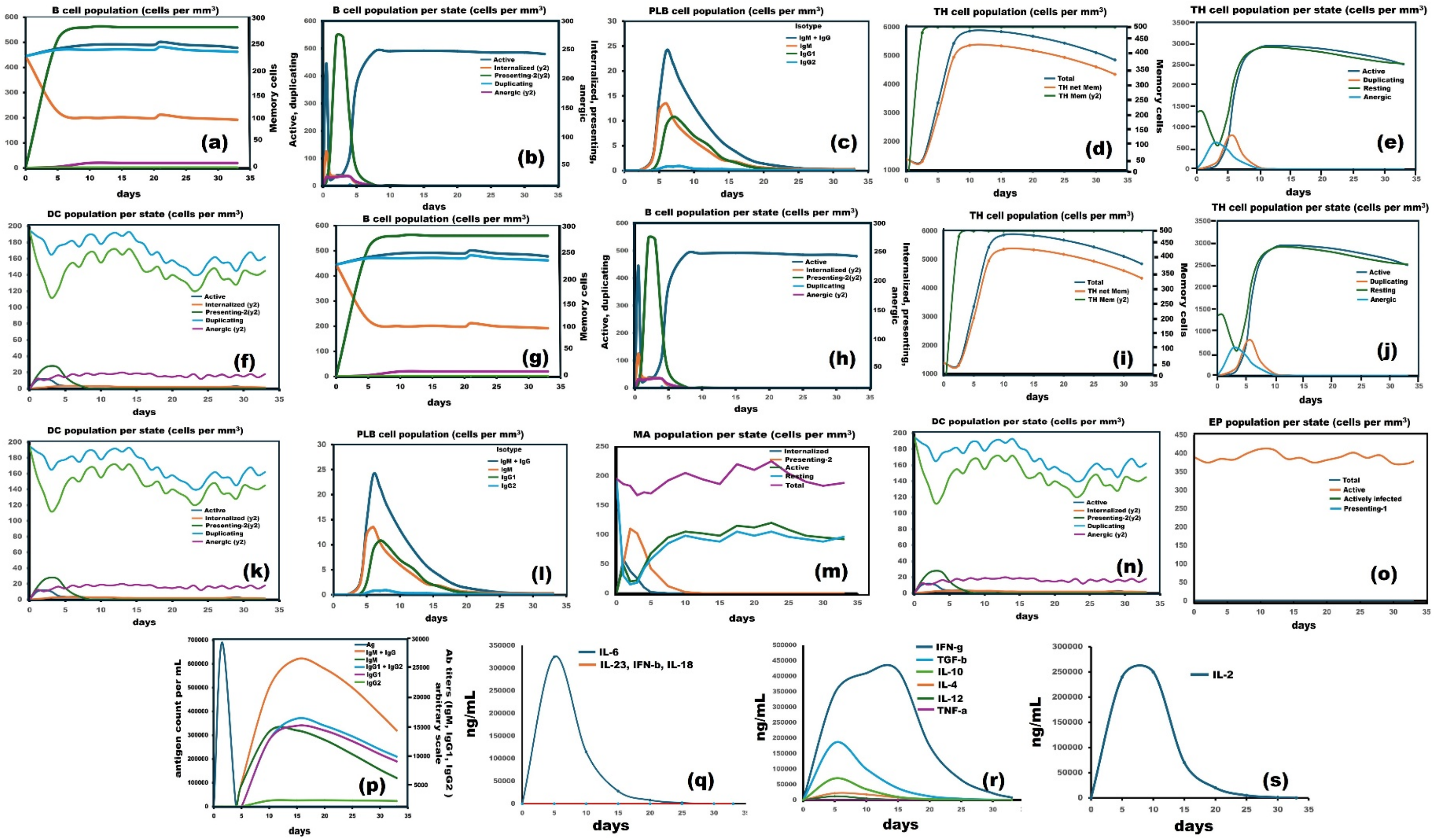

Supplementary Figure S5 (a-s). In silico immune simulation analysis of the multiepitope vaccine construct, The kinetics profile displaying antigen-immunoglobulin production (IgM, IgG1, IgG2, and IgM + IgG), B lymphocytes population per entity-state (i.e., showing counts for active, presenting on class-II, internalized the Ag, duplicating and anergic, B lymphocytes total count, memory cells, and sub-divided in isotypes IgM, IgG1 and IgG2, CD4 T-helper lymphocytes count sub-divided per entity-state (i.e., active, resting, anergic and duplicating), CD4 T-helper lymphocytes count. The plot shows total and memory counts, as well as CD4 T-regulatory lymphocyte count. Both total memory and per-entity-state counts are plotted: The CD8 T-cytotoxic lymphocytes count per entity-state, and the CD8 T-cytotoxic lymphocytes count. Total and memory shown, Dendritic cells. The DC can present antigenic peptides on both MHC class-I and class-II molecules, epithelial cells. The total count is broken down into active, virus-infected, and presented on class-I MHC molecule, Macrophages. Total count, internalized, presenting on MHC class-II, active and resting macrophages, Natural Killer cells (total count), Plasma B lymphocyte count subdivided per isotype (IgM, IgG1, and IgG2). The simulation was performed utilizing the antigen-combined sequence data from four proteins (NS1, NS2, VP1 and VP2) of the feline and canine parvovirus, and it was set to 100 with a volume of 10.
