# Supplementary material for: Immunoinformatics-Guided Computational Design and In Silico Validation of Multi-Epitope Vaccine Candidates Targeting Canine and Feline Parvoviruses
Source: Microorganisms. 2026 Aug 5;14(8):1721. doi: 10.3390/microorganisms14081721 (PMC13515720; doi:10.3390/microorganisms14081721)
Supplement: Supplementary file 1 [file microorganisms-14-01721-s001.zip › Supplementary Tables.pdf]

Supplementary Table S1: List of feline and canine parvovirus genome sequences retrieved from the NCBI GenBank database and used in this study. The table includes the GenBank accession number, virus/isolate name, country of origin, and collection date for each sequence.

S.No.	Accession Number	Virus	Country	collection date
1	JN033694	Canine parvovirus strain Laika-1993	Russia	1993
2	JQ268283	Canine parvovirus 2a strain CPV-LZ1	China: Lanzhou	3-Feb-11
3	JQ268284	Canine parvovirus 2b strain CPV-LZ2	China: Lanzhou	3-Feb-11
4	KP019621	Feline panleukopenia virus isolate TH091305	Thailand	16-Sep-13
5	KT382542	Canine parvovirus isolate CPV-SH14	China	20-Nov-14
6	KY073269	Canine parvovirus 2c isolate UFMT	Brazil	2015
7	MF805789	Canine parvovirus strain Canine/China/01/2016	China	2016
8	MF805790	Canine parvovirus strain Canine/China/02/2016	China	2016
9	MF805791	Canine parvovirus strain Canine/China/03/2016	China	2016
10	MF805792	Canine parvovirus strain Canine/China/04/2016	China	2016
11	MF805793	Canine parvovirus strain Canine/China/05/2016	China	2016
12	MF805794	Canine parvovirus strain Canine/China/06/2016	China	2016
13	MF805795	Canine parvovirus strain Canine/China/07/2016	China	2016
14	MF805796	Canine parvovirus strain Canine/China/08/2016	China	2016
15	MF805797	Canine parvovirus strain Canine/China/09/2016	China	2016
16	MF805798	Canine parvovirus strain Canine/China/10/2016	China	2016
17	MG013488	Canine parvovirus isolate CPV-SH1516	China	Oct-17
18	MH106698	Canine parvovirus isolate CPV-BJL1	China	Mar-15
19	MH106699	Canine parvovirus isolate CPV-BJL2	China	Sep-15
20	MH106700	Canine parvovirus isolate CPV-BJL3	China	Jan-16
21	MH476580	Canine parvovirus strain Canine/China/11/2017	China	2017
22	MH476581	Canine parvovirus strain Canine/China/12/2017	China	2017
23	MH476582	Canine parvovirus strain Canine/China/13/2017	China	2017

24	MH476583	Canine parvovirus strain Canine/China/14/2017	China	2017
25	MH476584	Canine parvovirus strain Canine/China/15/2017	China	2017
26	MH476585	Canine parvovirus strain Canine/China/16/2017	China	2017
27	MH476586	Canine parvovirus strain Canine/China/17/2017	China	2017
28	MH476587	Canine parvovirus strain Canine/China/18/2017	China	2017
29	MH476588	Canine parvovirus strain Canine/China/19/2017	China	2017
30	MH476589	Canine parvovirus strain Canine/China/20/2017	China	2017
31	MH476590	Canine parvovirus strain Canine/China/21/2017	China	2017
32	MH476591	Canine parvovirus strain Canine/China/22/2017	China	2017
33	MH476592	Canine parvovirus strain Canine/China/23/2017	China	2017
34	MH476593	Canine parvovirus strain Canine/China/24/2017	China	2017
35	MH660909	Canine parvovirus 2c strain 5 MGL	Mongolia	1-Mar-17
36	MN683826	Feline parvovirus isolate Fe-P2/2017	South Korea	2017
37	MT010564	Canine parvovirus isolate CPV-AHhf1	China	Aug-18
38	MT394163	Canine parvovirus isolate QD-12	China	2020
39	MT394164	Canine parvovirus isolate QD-15	China	2020
40	MT394165	Canine parvovirus isolate QD-202	China	2020
41	MT394166	Canine parvovirus isolate QD-201	China	2020
42	MT394167	Canine parvovirus isolate QD-20	China	2020
43	MT614366	Feline parvovirus isolate HF1	China	Apr-19
44	MT648202	Canine parvovirus isolate CPV-AHhf7	China	Jul-19
45	MT648203	Canine parvovirus isolate CPV-AHhf27	China	Jun-19
46	MT648204	Canine parvovirus isolate CPV-AHhf28	China	Jun-19
47	MT648205	Canine parvovirus isolate CPV-mas2	China	Apr-18
48	MT648206	Canine parvovirus isolate CPV-AHmas3	China	Jun-18
49	MT648207	Canine parvovirus isolate CPV-AHmas9	China	Oct-18
50	MT648208	Canine parvovirus isolate CPV-AHmas16	China	Nov-18
51	MT648209	Canine parvovirus isolate CPV-AHmas17	China	Nov-18
52	MT648210	Canine parvovirus isolate CPV-AHcf4	China	Sep-19

53	MT892649	Canine parvovirus isolate China-XY	China	Jun-19
54	MW653248	Canine parvovirus 2a strain CPV/Iran	Iran	02,02,2020
55	MW653255	Canine parvovirus strain CPV/8/Iran	Iran	02,02,2020
56	MW926314	Feline parvovirus isolate Cat 1	United Kingdom	2019
57	MW926315	Feline parvovirus isolate Cat 2	United Kingdom	2019
58	MW926316	Feline parvovirus isolate Cat 3	United Kingdom	2019
59	MZ362883	Feline parvovirus isolate AP. Roseworthy/SA/5371/20	Australia	2019
60	MZ742163	Feline parvovirus strain FPV-AUS-1	Australia	2017
61	MZ742164	Feline parvovirus strain FPV-AUS-2	Australia	2017
62	MZ742165	Feline parvovirus strain FPV-AUS-3	Australia	2017
63	MZ742166	Feline parvovirus strain FPV-AUS-4	Australia	2017
64	MZ742167	Feline parvovirus strain FPV-AUS-5	Australia	2017
65	MZ742168	Feline parvovirus strain FPV-AUS-6	Australia	2017
66	MZ742169	Feline parvovirus strain FPV-AUS-7	Australia	2017
67	MZ742170	Feline parvovirus strain FPV-AUS-8	Australia	2017
68	MZ742171	Feline parvovirus strain FPV-AUS-9	Australia	2017
69	MZ742172	Feline parvovirus strain FPV-AUS-10	Australia	2017
70	MZ742173	Feline parvovirus strain FPV-AUS-11	Australia	2017
71	MZ742174	Feline parvovirus strain FPV-AUS-12	Australia	2017
72	MZ742175	Feline parvovirus strain FPV-AUS-13	Australia	2017
73	MZ742176	Feline parvovirus strain FPV-AUS-14	Australia	2017
74	MZ742177	Feline parvovirus strain FPV-AUS-15	Australia	2017
75	MZ742178	Feline parvovirus strain FPV-AUS-16	Australia	2017
76	MZ742179	Feline parvovirus strain FPV-AUS-17	Australia	2017
77	MZ742180	Feline parvovirus strain FPV-AUS-18	Australia	2017
78	MZ913313	Feline parvovirus strain F-18	China	2021
79	MZ913314	Feline parvovirus strain D13	China	2021
80	MZ913315	Feline parvovirus strain C-F9	China	2019
81	MZ913316	Feline parvovirus strain C-F88	China	2019

82	MZ913317	Feline parvovirus strain C-1	China	2019
83	MZ913318	Feline parvovirus strain C-4	China	2019
84	MZ913319	Feline parvovirus strain C-9	China	2019
85	NC_001539	Canine parvovirus	USA	2000
86	OK128324	Feline parvovirus strain C-DY6	China	2019
87	OK128325	Feline parvovirus strain D7	China	2021
88	OL547731	Feline parvovirus strain F-C	China	2021
89	OL547732	Feline parvovirus strain F-D	China	2020
90	OL547733	Feline parvovirus strain F-D35	China	2019
91	OL547734	Feline parvovirus strain F-E	China	2020
92	OL547735	Feline parvovirus strain F-D34	China	2019
93	OL547736	Feline parvovirus strain F-D33	China	2019
94	OL547737	Feline parvovirus strain F-A	China	2020
95	OP093952	Canine parvovirus strain CPV/PK06/2019/BRA	Brazil	2019
96	OP093953	Canine parvovirus strain CPV/PK05/2019/BRA	Brazil	2019
97	OP093954	Canine parvovirus strain CPV/PK04/2019/BRA	Brazil	2019
98	OP093955	Canine parvovirus strain CPV/PK03/2019/BRA	Brazil	2019
99	OP093956	Canine parvovirus strain CPV/PK02/2019/BRA	Brazil	2019
100	OP210300	Canine parvovirus isolate Par2-2022	Viet Nam	2022
101	OP957419	Canine parvovirus isolate CPV-T11	China	2016
102	OP985292	Canine parvovirus strain RCD-PrV/BJF6/red-collared dove/2018	China	11-Dec-19
103	OQ366402	Canine parvovirus isolate Turkey_Ankara_2	Turkey: Ankara	2-Jan-21
104	OQ366403	Canine parvovirus isolate Turkey_Samsun_3	Turkey: Samsun	16-Feb-21
105	OQ366404	Canine parvovirus isolate Turkey_Sanliurfa_3	Turkey: Sanliurfa	19-Oct-21
106	OQ366405	Canine parvovirus isolate Turkey_Izmir_2	Turkey: Izmir	30-Aug-20
107	OQ718429	Feline panleukopenia virus isolate FPV/NS1/cat/IT	Italy	2021
108	OR198066	Feline parvovirus strain NTU/C148/2020	Taiwan	2020
109	OR230510	Canine parvovirus 2a strain CPV/UFT01/2022/BRA	Brazil	2022/2023
110	OR230511	Canine parvovirus 2a strain CPV/UFT02/2022/BRA	Brazil	2022/2023

111	OR230512	Canine parvovirus 2a strain CPV/UFT03/2022/BRA	Brazil	2022/2023
112	OR230513	Canine parvovirus 2a strain CPV/UFT04/2022/BRA	Brazil	2022/2023
113	OR230514	Canine parvovirus 2a strain CPV/UFT05/2022/BRA	Brazil	2022/2023
114	OR230515	Canine parvovirus 2a strain CPV/UFT06/2022/BRA	Brazil	2022/2023
115	OR230516	Canine parvovirus 2a strain CPV/UFT07/2022/BRA	Brazil	2022/2023
116	OR257444	Feline parvovirus isolate WH 1	China	2018
117	OR257445	Feline parvovirus isolate M1	China	2022
118	OR257446	Feline parvovirus isolate HY	China	2021
119	OR451707	Canine parvovirus 2 isolate YDI1	Iraq	3-Feb-22
120	OR528745	Canine parvovirus isolate CPV_EC113_2a	Ecuador	2012
121	OR528746	Canine parvovirus isolate CPV_86_2c	Uruguay	2007
122	OR528747	Canine parvovirus isolate CPV_EC116_2c	Ecuador	2012
123	OR528748	Canine parvovirus isolate CPV_ARG_13_2c	Argentina	2010
124	OR528749	Canine parvovirus isolate CPV_ARG22_2b	Argentina	2013
125	OR528750	Canine parvovirus isolate CPV_PE3_2b	Peru	2017
126	OR528751	Canine parvovirus isolate CPV_PE_34_2a	Peru	2019
127	OR528752	Canine parvovirus isolate CPV_PE1_2c	Peru	2017
128	OR528753	Canine parvovirus isolate CPV_Uy6_2a	Uruguay	2006
129	OR528754	Canine parvovirus isolate CPV_UY_252_2a	Uruguay	2010
130	OR528755	Canine parvovirus isolate CPV2_Vac(NL_35_D)	Uruguay	2023
131	OR667800	Canine parvovirus 2 isolate YDI2	Iraq	3-Feb-22
132	OR667801	Canine parvovirus 2 isolate YDI3	Iraq	3-Apr-22
133	OR667802	Canine parvovirus 2 isolate YDI4	Iraq	13-Apr-22
134	OR667803	Canine parvovirus 2 isolate YDI5	Iraq	18-Jun-22
135	OR667804	Canine parvovirus 2 isolate YDI6	Iraq	18-Jun-22
136	OR783315	Feline parvovirus strain LZ19/2022	China	2022
137	PP973493	Feline parvovirus isolate FPV JL 2280	China	Aug-22
138	PV784947	Canine parvovirus isolate CPV-KZ-2024	Kazakhstan: Almaty	2024-11
139	M19296	Canine parvovirus strain CPV-N	USA	2-Feb-88

140	M38245	Canine parvovirus	USA	27-Aug-90
141	D26079	Canine parvovirus	Japan	1993
142	MZ647470	Canine parvovirus strain MO	USA	2017
143	MF416372	Canine parvovirus 2 isolate NYC/Bronx/poolP16-17	USA	2015

Supplementary Table S2: shows the VP1, VP2, NS1 and NS2 gene sequences and their corresponding conserved regions identified through multiple sequence alignment. The conserved regions represent highly preserved amino acid sequences across the analyzed strains and were selected for subsequent epitope prediction and vaccine design analyses.

>AAB02799 capsid protein VP1 [Canine parvovirus]
MAPPAKRARRGLVPPGYKYLGPGNSLDQGEPTNPSDAAAKEHDEAYAAYLRSGKNPYLYFSPADQRFIDQTKDAKDWGGKIGHYFFRAKKAI<br>APVLTDTPDHPSTSRPTKPTKRSKPPPHIFINLAKKKKAGAGQVKRDNLAPMSDGAVQPDGGQPAVRNERATGSGNGSGGGGGGGSGGVGIST<br>GTFNNQTEFKFLENGWVEITANSSRLVHLNMPESENYRRVVVNNMDKTAVNGNMALDDIHAQIVTPWSLVDANAWGVWFNPGDWQLIVN<br>TMSELHLVSFEQEIFNVVLKTVSESATQPPTKVYNNDLTASLMVALDSNNTMPFTPAAMRSETLGFYPWKPTIPTPWRYYFQWDRTLIPSHTGTS<br>GTPTNIYHGTDPDDVQFYTIENSVPVHLLRTGDEFATGTFFFDCKPCRLTHTWQTNRALGLPPFLNSLPQSEGATNFGDIGVQQDKRRGVTQM<br>GNTNYITEATIMRPAEVGYSAPYYSFEASTQGPFKTPIAAGRGGAQTDENQAADGNPRYAFGRQHGQKTTTTGETPERFTYIAHQDTGRYPEGD<br>WIQNINFNLPVTNDNVLLPTDPIGGKTGINYTNIFNTYGPLTALNNVPPVYPNGQIWDKEFDTDLKPRLHVNAPFVCQNNCPGQLFVKVAPNL<br>TNEYDPDASANMSRIVTYSDFWWKGKLVFKAKLRASHTWNPIQQMSINVDNQFNYVPSNIGGMKIVYEKSQLAPRKLY
>QOR32241 VP1 [Feline parvovirus]
MAPPAKRARRGLVPPGYKYLGPGNSLDQGEPTNPSDAAAKEHDEAYAAYLRSGKNPYLYFSPADQRFIDQTKDAKDWGGKIGHYFFRAKKAI<br>APVLTDTPDHPSTSRPTKPTKRSKPPPHIFINLAKKKKAGAGQVKRDNLAPMSDGAVQPDGGQPAVRNERATGSGNGSGGGGGGGSGGVGIST<br>GTFNNQTEFKFLENGWVEITANSSRLVHLNMPESENYKRVVVNNMDKTSVKGNMALDDTHVQIVTPWSLVDANAWGVWFNPGDWQLIVN<br>TMSELHLVSFEQEIFNVVLKTVSESATQPPTKVYNNDLTASLMVALDSNNTMPFTPAAMRSETLGFYPWKPTIPTPWRYYFQWDRTLIPSHTGTS<br>GTPTNVYHGTDPDDVQFYTIENSVPVHLLRTGDEFATGTFFFDCKPCRLTHTWQTNRALGLPPFLNSLPQSEGATNFGDIGVQQDKRRGVTQM<br>GNTDYITEATIMRPAEVGYSAPYYSFEASTQGPFKTPIAAGRGGAQTDENQAADGDPRYAFGRQHGQKTTTTGETPERFTYIAHQDTGRYPEGD<br>WIQNINFNLPVTNDNVLLPTDPIGGKTGINYTNIFNTYGPLTALNNVPPVYPNGQIWDKEFDTDLKPRLHVNAPFVCQNNCPGQLFVKVAPNL<br>TNEYDPDASANMSRIVTYSDFWWKGKLVFKAKLRASHTWNPIQQMSINVDNQFNYVPNNIGAMKIVYEKSQLAPRKLY
**Conserved region: VP1**
**MAPPAKRARRGLVPPGYKYLGPGNSLDQGEPTNPSDAAAKEHDEAYAAYLRSGKNPYLYFSPADQRFIDQTKDAKDWGGKIGHYFFRA<br>KKAIAPVLTDTPDHPSTSRPTKPTKRSKPPPHIFINLAKKKKAGAGQVKRDNLAPMSDGAVQPDGGQPAVRNERATGSGNGSGGGGG<br>GGSGGVGISTGTFNNQTEFKFLENGWVEITANSSRLVHLNMPESENYRVVVNNMDKTVNGNMALDDIHQIVTPWSLVDANAWGVWF<br>NPGDWQLIVNTMSELHLVSFEQEIFNVVLKTVSESATQPPTKVYNNDLTASLMVALDSNNTMPFTPAAMRSETLGFYPWKPTIPTPWRYY<br>FQWDRTLIPSHTGTSGTPTNYHGTDPDDVQFYTIENSVPVHLLRTGDEFATGTFFFDCKPCRLTHTWQTNRALGLPPFLNSLPQSEGATNF<br>GDIGVQQDKRRGVTQMGNTYITEATIMRPAEVGYSAPYYSFEASTQGPFKTPIAAGRGGAQTDENQAADGPRYAFGRQHGQKTTTTGE<br>TPERFTYIAHQDTGRYPEGDWIQNINFNLPVTNDNVLLPTDPIGGKTGINYTNIFNTYGPLTALNNVPPVYPNGQIWDKEFDTDLKPRLH<br>VNAPFVCQNNCPGQLFVKVAPNLTNEYDPDASANMSRIVTYSDFWWKGKLVFKAKLRASHTWNPIQQMSINVDNQFNYVPNIGMKIV<br>YEKSQLAPRKLY**

>AAY99064 VP2 [Canine parvovirus]
MSDGAVQPDGGQPAVRNERATGSGNGSGGGGGGGSGGVGISTGTFNNQTEFKFLENGWVEITANSSRLVHLNMPESENYRRVVVNNLDKTA<br>VNGNMALDDTHAQIVTPWSLVDANAWGVWFNPGDWQLIVNTMSELHLVSFEQEIFNVVLKTVSESATQPPTKVYNNDLTASLMVALDSNNT<br>MPFTPAAMRSETLGFYPWKPTIPTPWRYYFQWDRTLIPSHTGTSGTPTNIYHGTDPDDVQFYTIENSVPVHLLRTGDEFATGTFFFDCKPCRLTH<br>TWQTNRALGLPPFLNSLPQAEGGTNFGYIGVQQDKRRGVTQMGNTNYITEATIMRPAEVGYSAPYYSFEASTQGPFKTPIAAGRGGAQTDENQ<br>AADGDPRYAFGRQHGQKTTTTGETPERFTYIAHQDTGRYPEGDWIQNINFNLPVTNDNVLLPTDPIGGKAGINYTNIFNTYGPLTALNNVPPV<br>YPNGQIWDKEFDTDLKPRLHVNAPFVCQNNCPGQLFVKVAPNLTNEYDPDASANMSRIVTYSDFWWKGKLVFKAKLRASHTWNPIQQMSIN<br>VDNQFNYVPSNIGGMKIVYEKSQLAPRKLY
>ADN18774 VP2 protein [Feline parvovirus]
MSDGAVQPDGGQPAVRNERATGSGNGSGGGGGGGSGGVGISTGTFNNQTEFKFLENGWVEITANSSRLVHLNMPESENYKRVVVNNMDKTA<br>VKGNMALDDIHVQIVTPWSLVDANAWGVWFNPGDWQLIVNTMSELHLVSFEQEIFNVVLKTVSESATQPPTKVYNNDLTASLMVALDSNNT<br>MPFTPAAMRSETLGFYPWKPTIPTPWRYYFQWDRTLVPSHTGTSGTPTNIYHGTDPDDVQFYTIENSVPVHLLRTGDEFATGTFFFDCKPCRLTH<br>TWQTNRALGLPPFLNSLPQSEGPTNFGDIGVQQDKRRGVTQMGNTDYITEATIMRPAEVGYSAPYYSFEASTQGPFKTPIAAGRGGAQTDENQ<br>AADGDPRYAFGRQHGQKTTTTGETPERFTYIAHQDTGRYPAGDWIQNINFNLPVTNDNVLLPTDPIGGKTGINYTNIFNTYGPLTALNNVPPV<br>YPNGQIWDKEFDTDLKPRLHVNAPFVCQNNCPGQLFVKVAPNLTNEYDPDASANMSRIVTYSDFWWKGKLVFKAKLRASHTWNPIQQMSIN<br>VDNQFNYLPNNIGAMKIVYEKSQLAPRKLY
**Conserved region:VP2**
**MSDGAVQPDGGQPAVRNERATGSGNGSGGGGGGGSGGVGISTGTFNNQTEFKFLENGWVEITANSSRLVHLNMPESENYRVVVNND<br>KTAVNGNMALDDHQIVTPWSLVDANAWGVWFNPGDWQLIVNTMSELHLVSFEQEIFNVVLKTVSESATQPPTKVYNNDLTASLMVAL<br>DSNNTMPFTPAAMRSETLGFYPWKPTIPTPWRYYFQWDRTLPSHTGTSGTPTNIYHGTDPDDVQFYTIENSVPVHLLRTGDEFATGTFFF<br>DCKPCRLTHTWQTNRALGLPPFLNSLPQEGGTNFGYIGVQQDKRRGVTQMGNTYITEATIMRPAEVGYSAPYYSFEASTQGPFKTPIAAG<br>RGGAQTDENQAADGDPRYAFGRQHGQKTTTTGETPERFTYIAHQDTGRYPEGDWIQNINFNLPVTNDNVLLPTDPIGGKGINYTNIFN<br>TYGPLTALNNVPPVYPNGQIWDKEFDTDLKPRLHVNAPFVCQNNCPGQLFVKVAPNLTNEYDPDASANMSRIVTYSDFWWKGKLVFKA<br>KLRASHTWNPIQQMSINVDNQFNYVPNIGGMKIVYEKSQLAPRKLY**
>QOR32239 NS1 [Feline parvovirus]
MSGNQYTEEVMEGVNWLKKHAEDEAFSFVFKCDNVQLNGKDVRWNNYTKPIQNEELTSLIRGAQTAMDQTEEEEMDWESEVDSLAKKQVQ<br>TFDALIKKCLFEVFVSKNIEPNECVWFIQHEWGKDQGWHCHVLLHSKNLQQATGKWLRRQMNMYWSRWLVTLCSVNLTPTEKIKLREIAEDS<br>EWVTILTYRHKQTKKDYVKMVHFGNMIAYYFLTKKKIVHMTKESGYFLSTDSGWKFNFMKYQDRHTVSTLYTEQMKPETVETTVTTAQETKRG<br>RIQTKKEVSIKCTLRDLVSKRVTSPEDWMMLQPDSYIEMMAQPGGENLLKNTLEICTLTLARTKTAFELILEKADNTKLTNFDLANSRTCQIFRM<br>HGWNWIKVCHAIACVLNRQGGKRNTVLFHGPASTGKSIIAQAIAQAVGNVGCYNAANVNFPFNDCTNENLIWIEEAGNFGQQVNQFKAICS<br>GQTIRIDQKGKGSKQIEPTPVIMTTNENITIVRIGCEERPEHTQPIRDRMLNIKLVCKLPGDFGLVDKEEWPLICAWLVKHGYESTMANYTHHWG

KVPEWDENWAEPKIQEGINSPGCKDLETQAASNPQSQDQLLTPLTPDVVDLALEPWSTPDTPIAETANQQSNQLGVTHKDVQASPTWSEIEAD<br>LRAIFTSEQLEEDFRDDLD
>AAB02798 NS1 protein [Canine parvovirus]
MSGNQYTEEVMEGVNWLKKHAENEAFSFVFKCDNVQLNGKDVRWNNYTKPIQNEELTSLIRGAQTAMDQTEEEEMDWESEVDSLAKKQVQ<br>TFDALIKKCLFEVFVSKNIEPNECVWFIQHEWGKDQGWHCHVLLHSKNLQQATGKWLRRQMNMYWSRWLVTLCSVNLTPTEKIKLREIAEDS<br>EWVTILTYRHKQTKKDYVKMVHFGNMIAYYFLTKKKIVHMTKESGYFLSTDSGWKFNFMKYQDRQIVSTLYTEQMKPETVETTVTTAQETKRG<br>RIQTKKEVSIKCTLRDLVSKRVTSPEDWMMLQPDSYIEMMAQPGGENLLKNTLEICTLTLARTKTAFELILEKADNTKLTNFDLANSRTCQIFRM<br>HGWNWIKVCHAIACVLNRQGGKRNTVLFHGPASTGKSIIAQAIAQAVGNVGCYNAANVNFPFNDCTNKNLIWIEEAGNFGQQVNQFKAICS<br>GQTIRIDQKGKGSKQIEPTPVIMTTNENITIVRIGCEERPEHTQPIRDRMLNIKLVCKLPGDFGLVDKEEWPLICAWLVKHGYESTMANYTHHWG<br>KVPEWDENWAEPKIQEGINSPGCKDLETQAASNPQSQDQVLTPLTPDVVDLALEPWSTPDTPIAETANQQSNQLGVTHKDVQASPTWSEIEAD<br>LRAIFTSEQLEEDFRDDLD
**Conserved region: NS1**
**MSGNQYTEEVMEGVNWLKKHAEEAFSFVFKCDNVQLNGKDVRWNNYTKPIQNEELTSLIRGAQTAMDQTEEEEMDWESEVDSLAKK<br>QVQTFDALIKKCLFEVFVSKNIEPNECVWFIQHEWGKDQGWHCHVLLHSKNLQQATGKWLRRQMNMYWSRWLVTLCSVNLTPTEKIK<br>LREIAEDSEWVTILTYRHKQTKKDYVKMVHFGNMIAYYFLTKKKIVHMTKESGYFLSTDSGWKFNFMKYQDRHTVSTLYTEQMKPETVE<br>TTVTTAQETKRGRIQTKKEVSIKCTLRDLVSKRVTSPEDWMMLQPDSYIEMMAQPGGENLLKNTLEICTLTLARTKTAFELILEKADNTKL<br>TNFDLANSRTCQIFRMHGWNWIKVCHAIACVLNRQGGKRNTVLFHGPASTGKSIIAQAIAQAVGNVGCYNAANVNFPFNDCTNENLI<br>WIEEAGNFGQQVNQFKAICSGQTIRIDQKGKGSKQIEPTPVIMTTNENITIVRIGCEERPEHTQPIRDRMLNIKLVCKLPGDFGLVDKEEW<br>PLICAWLVKHGYESTMANYTHHWGKVPEWDENWAEPKIQEGINSPGCKDLETQAASNPQSQDQLLTPLTPDVVDLALEPWSTPDTPIAE<br>TANQQSNQLGVTHKDVQASPTWSEIEADLRAIFTSEQLEEDFRDDLD**
>AAV54173 NS2 [Canine parvovirus]
MSGNQYTEEVMEGVNWLKKHAENEAFSFVFKCDNVQLNGKDVRWNNYTKPIQNEELTSLIRGAQTAMDQTEEEEMDWESEVDSLAKKLQR<br>LRDTSGKQSSESRPSSNSSDSGRSGPCTGTVEYSRYAYCRNCKSTIKPTWRYSQRRASESDMVRNRGRPESHLYF
>QOR32240 NS2 [Feline parvovirus]
MSGNQYTEEVMEGVNWLKKHAEDEAFSFVFKCDNVQLNGKDVRWNNYTKPIQNEELTSLIRGAQTAMDQTEEEEMDWESEVDSLAKKLQR<br>LRDTSGKQSSESRPTSNSSDSGRSGPCTGTVEYSRYAYCRNCKSTIKPTWRYSQRRASESDVVRNRGRPESHLYF
**Conserved region: NS2**
**MSGNQYTEEVMEGVNWLKKHAEEAFSFVFKCDNVQLNGKDVRWNNYTKPIQNEELTSLIRGAQTAMDQTEEEEMDWESEVDSLAKKL<br>QRLRDTSGKQSSESRPSNSSDSGRSGPCTGTVEYSRYAYCRNCKSTIKPTWRYSQRRASESDVRNRGRPESHLYF**

Supplementary Table S3 shows the Predicted conformational (discontinuous) B-cell epitopes of the VP1 protein identified using the ElliPro server. Predicted epitopes were mapped onto the three-dimensional structure of VP1 based on structural protrusion analysis. Higher protrusion index (PI) values indicate greater surface exposure and antigenic potential, suggesting an increased likelihood of antibody recognition.

No.	Residues	Number of residues	Score	3D structure with solvent accessibility
1	A:M1, A:A2, A:P3, A:P4, A:A5, A:K6	6	0.948	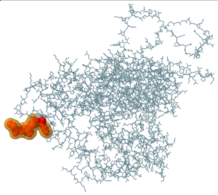
2	A:A522, A:F523, A:G524, A:R525, A:Q526, A:H527, A:G528, A:Q529, A:K530, A:T531, A:T532, A:T533, A:T534, A:G535, A:E536, A:T537, A:P538, A:E539, A:R540	19	0.935	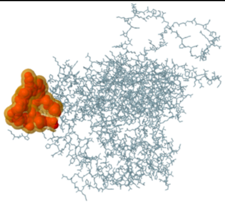
3	A:D139, A:N140, A:L141, A:A142, A:P143, A:M144, A:S145, A:D146, A:G147, A:A148, A:V149, A:Q150, A:P151, A:D152, A:G153, A:G154, A:Q155, A:P156, A:A157, A:V158, A:R159, A:N160, A:E161	23	0.9	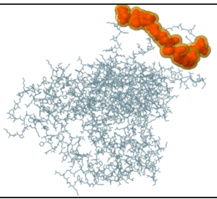
4	A:R107, A:P108, A:T109, A:K110, A:P111, A:T112, A:K113	7	0.844	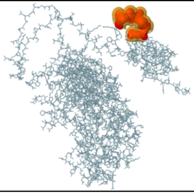
5	A:R162, A:A163, A:T164, A:G165, A:S166, A:G167, A:N168, A:G169, A:S170, A:G171, A:G172, A:G173, A:G174, A:G175, A:G176, A:G177, A:S178, A:G179, A:G180, A:V181, A:G182, A:I183, A:S184, A:T185	24	0.818	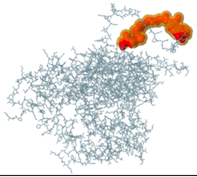

6	A:R426, A:G429, A:L430, A:P431, A:P432, A:F433, A:L434, A:N435, A:S436, A:L437, A:P438, A:Q439, A:S440, A:E441, A:G442, A:A443, A:T444, A:N445, A:F446, A:G447, A:D448, A:I449, A:G450, A:V451, A:Q452, A:Q453, A:D454, A:K455, A:R456, A:G458, A:V459, A:T460, A:Q461, A:M462, A:G463, A:N464, A:T465, A:N466, A:Y467, A:I468, A:T469, A:E470, A:A471, A:T472, A:I473	45	0.76	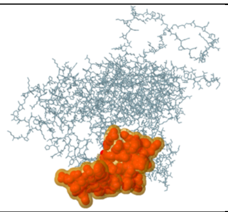
7	A:R7, A:A8, A:R10, A:G11, A:L12, A:V13, A:P14, A:P15, A:G16, A:Y17, A:K18, A:Y19, A:L20, A:G21, A:P22, A:G23, A:N24, A:S25, A:L26, A:D27, A:Q28, A:G29, A:E30, A:P31, A:T32, A:N33, A:P34, A:S35, A:D36, A:A37, A:A38, A:A39, A:K40, A:E41, A:H42, A:D43, A:E44, A:A45, A:Y46, A:A47, A:A48, A:Y49, A:L50, A:R51, A:S52, A:G53, A:K54, A:N55, A:P56, A:Y57, A:L58, A:Y59, A:F60, A:S61, A:P62, A:A63, A:D64, A:Q65, A:R66, A:F67, A:I68, A:D69, A:Q70, A:T71, A:K72, A:D73, A:A74, A:K75, A:D76, A:W77, A:G78, A:G79, A:K80, A:H83, A:K90, A:A93, A:P94, A:V95, A:L96, A:T97, A:D98, A:T99, A:P100, A:D101, A:H102, A:P103, A:S104, A:T105, A:S106	89	0.726	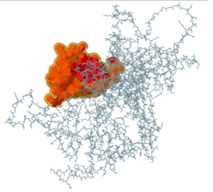
8	A:A131, A:G132, A:A133, A:G134, A:Q135, A:V136, A:K137	7	0.717	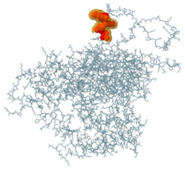
9	A:Y653, A:D654, A:P655, A:D656, A:A657, A:S658, A:A659, A:N660	8	0.709	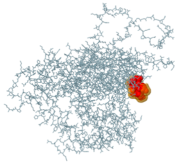
10	A:S487, A:F488, A:E489, A:A490, A:S491, A:T492, A:Q493, A:G494, A:P495, A:F496, A:K497, A:T498	12	0.708	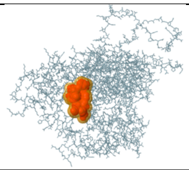
11	A:T542, A:Y543, A:I544, A:A545, A:H546, A:Q547, A:D548, A:T549	8	0.647	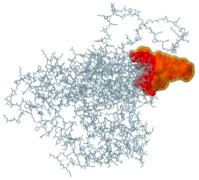

12	A:M216, A:T295, A:V296, A:S297, A:E298, A:S299, A:A300, A:T301, A:Q302, A:P303, A:P304, A:T305, A:K306, A:V307, A:Y308, A:N309, A:N310, A:D311, A:L312, A:T313, A:T400, A:P647, A:N648, A:L649, A:T650, A:N651, A:E652, A:M661, A:S662, A:R663, A:I664, A:V665	32	0.646	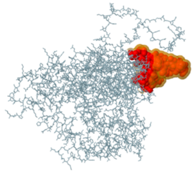
13	A:A502, A:G503, A:R504, A:G505, A:G506, A:A507, A:Q508, A:T509, A:D510, A:E511, A:N512, A:Q513, A:A514, A:A515, A:D516, A:G517, A:N518, A:P519, A:Y521	19	0.635	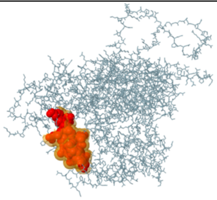
14	A:N228, A:N229, A:M230, A:D231, A:K232, A:T233, A:A234, A:V235, A:N236, A:G237, A:N238, A:M239, A:D242, A:I244, A:Q356, A:W357, A:D358, A:T360, A:L361, A:I362, A:P363, A:S364, A:H365, A:T366, A:G367, A:T368, A:S369, A:G370, A:T371, A:P372, A:T373, A:N374, A:I375, A:Y376, A:H377, A:G378, A:T379, A:D380, A:P381, A:D382, A:N560, A:I561, A:N562, A:F563, A:N564, A:L565, A:P566, A:V567, A:T568, A:N569, A:D570, A:V572, A:L573, A:L574, A:P575, A:T576, A:D577, A:P578, A:I579, A:G580, A:G581, A:K582, A:T583, A:G584, A:I585, A:N586, A:Y587, A:T588	68	0.628	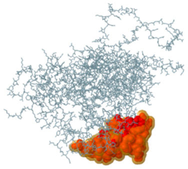
15	A:Y552, A:P553, A:E554	3	0.52	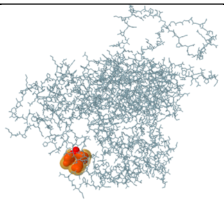
16	A:S707, A:N708, A:I709, A:G710	4	0.517	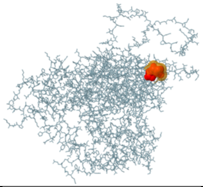
17	A:N697, A:V698, A:D699, A:Q701	4	0.513	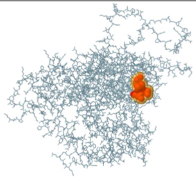

Supplementary Table S4 shows the Predicted conformational (discontinuous) B-cell epitopes of the VP2 protein identified using the ElliPro server and predicted epitopes were mapped onto the three-dimensional structure of VP2 based on structural protrusion analysis

No.	Residues	Number of residues	Score	3D structure with solvent accessibility
1	A:S2, A:D3, A:G4, A:A5, A:V6, A:Q7, A:P8, A:D9, A:G10, A:G11, A:Q12, A:P13, A:A14, A:V15, A:N17	15	0.904	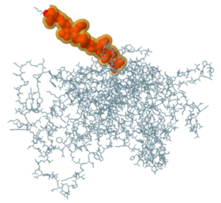
2	A:R19, A:A20, A:T21, A:G22, A:S23, A:G24, A:N25, A:G26, A:S27, A:G28, A:G29, A:G30, A:G31, A:G32, A:G33, A:G34, A:S35, A:G36, A:G37, A:V38, A:G39, A:I40, A:S41, A:T42, A:G43, A:T44	26	0.839	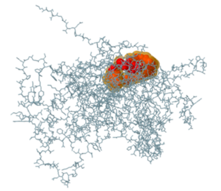
3	A:Y502, A:D503, A:P504, A:D505, A:A506, A:S507, A:A508, A:N509	8	0.825	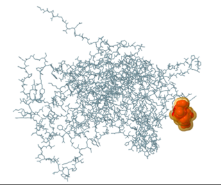
4	A:W407, A:I408, A:Q409, A:N410, A:I411, A:N412, A:F413, A:N414, A:L415, A:P416, A:V417, A:T418, A:N419, A:D420, A:N421, A:V422, A:L423, A:L424, A:P425, A:T426, A:D427, A:P428, A:I429, A:G430, A:G431, A:K432, A:G433, A:I434, A:N435, A:Y436	30	0.823	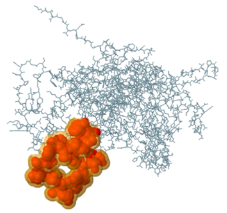
5	A:Q275, A:T276, A:R278, A:A279, A:L280, A:G281, A:L282, A:P283, A:P284, A:F285, A:L286, A:N287, A:S288, A:L289, A:P290, A:Q291, A:G293, A:G294, A:T295, A:N296, A:F297, A:G298, A:Y299, A:I300, A:G301, A:V302, A:Q303, A:Q304, A:D305, A:K306, A:R307, A:R308, A:G309, A:V310, A:T311, A:Q312, A:M313, A:G314, A:N315, A:T316, A:Y317, A:I318, A:T319, A:E320, A:A321, A:T322, A:I323, A:M324, A:R325, A:P326, A:A327	51	0.785	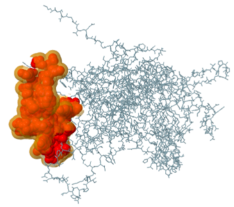

6	A:M73, A:P74, A:E75, A:S76, A:E77, A:N78, A:Y79, A:R80, A:P104, A:L146, A:K147, A:T148, A:V149, A:S150, A:E151, A:S152, A:A153, A:T154, A:Q155, A:P156, A:P157, A:T158, A:K159, A:V160, A:Y161, A:N162, A:N163, A:D164, A:L165, A:T166, A:A167, A:S168, A:L249, A:R251, A:T252, A:G253, A:D254, A:E255, A:A495, A:P496, A:N497, A:L498, A:T499, A:N500, A:E501, A:M510, A:S511, A:R512, A:I513, A:V514, A:T515, A:Y516	52	0.668	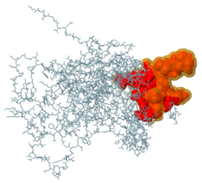
7	A:V81, A:V82, A:V83, A:N84, A:N85, A:D86, A:K87, A:T88, A:A89, A:V90, A:N91, A:G92, A:N93, A:M94, A:A95, A:L96, A:D97, A:D98, A:H99, A:F208, A:Q209, A:W210, A:D211, A:R212, A:T213, A:L214, A:P215, A:S216, A:H217, A:T218, A:G219, A:T220, A:S221, A:G222, A:T223, A:P224, A:T225, A:N226, A:I227, A:Y228, A:H229, A:G230, A:T231, A:D232, A:P233, A:D234, A:D235, A:V236, A:Q237, A:F238, A:Y239, A:T240, A:E242, A:N243, A:S244, A:V245, A:E339, A:A340, A:S341, A:T342, A:Q343, A:G344, A:P345	63	0.667	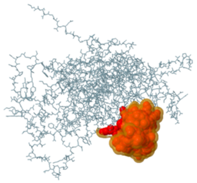
8	A:K52, A:F53, A:L54, A:E55, A:N56, A:G57, A:W58, A:L531	8	0.621	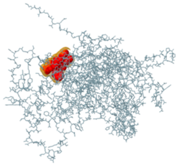
9	A:R532, A:A533, A:S534, A:H535	4	0.597	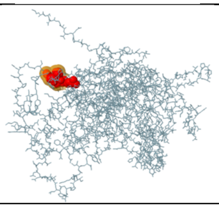
10	A:Q379, A:K380, A:T381, A:T382, A:T383, A:T384, A:G385, A:E386, A:T387, A:M543, A:S544, A:I545, A:N546, A:V547, A:D548, A:N549, A:Q550, A:F551, A:N552, A:V554, A:P555, A:N556, A:I557, A:G558, A:G559, A:M560, A:K561	27	0.594	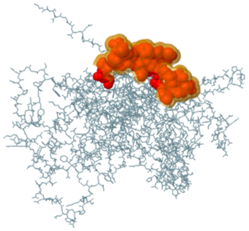

11	A:T536, A:W537, A:N538, A:P539, A:I540, A:Q541	6	0.573	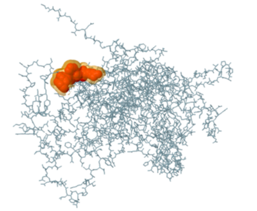
12	A:G355, A:G356, A:A357, A:Q358, A:T359, A:D360, A:E361, A:N362, A:Q363	9	0.555	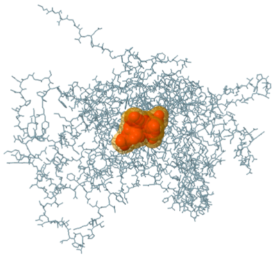
13	A:T437, A:N438, A:I439, A:F440, A:N441	5	0.518	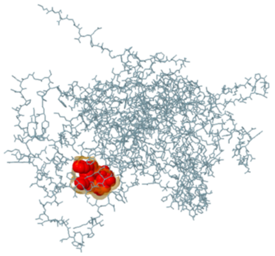

Supplementary Table S5 shows the Predicted Conformational (discontinuous) B-cell epitopes of the NS1 protein identified using the ElliPro server and predicted epitopes were mapped onto the three-dimensional structure of NS1 based on structural protrusion analysis

No.	Residues	Number of residues	Score	3D structure with solvent accessibility
1	A:L596, A:L597, A:T598, A:P599, A:L600, A:T601, A:P602, A:D603, A:V604, A:V605, A:D606, A:L607, A:A608, A:L609, A:E610, A:P611	16	0.934	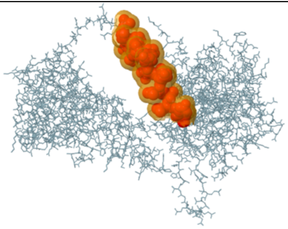
2	A:S613, A:T614, A:P615, A:D616, A:T617, A:P618, A:I619, A:A620, A:E621, A:T622, A:A623, A:N624, A:Q625, A:Q626, A:S627, A:N628, A:Q629, A:L630, A:G631, A:V632, A:T633, A:H634	22	0.929	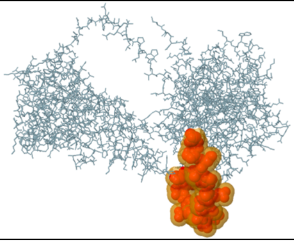
3	A:D665, A:D666, A:L667, A:D668	4	0.878	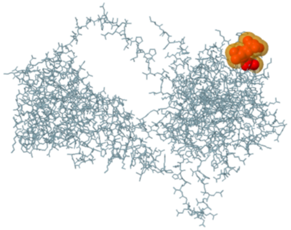
4	A:T264, A:T265, A:V266, A:T267, A:T268, A:A269, A:Q270, A:E271, A:T272, A:K273	10	0.783	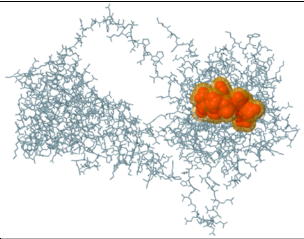

5	A:L354, A:T355, A:N356, A:F357, A:D358, A:L359, A:A360, A:N361, A:S362, A:R363, A:C365, A:Q366, A:I367, A:F368, A:M370, A:H371, A:G372, A:W373, A:N374, A:W375, A:I376, A:K377, A:K392, A:R393, A:H399, A:G400, A:P401, A:A402, A:S403, A:T404, A:G405, A:K406, A:I408, A:E444, A:E445, A:A446, A:G447, A:N448, A:F449, A:G450, A:Q451, A:Q452, A:V453, A:N454, A:K457, A:T486, A:N487, A:E488, A:N489, A:I490, A:T491, A:I492, A:V493, A:R494, A:I495, A:G496, A:C497, A:E498, A:E499, A:R500, A:P501, A:E502, A:H503, A:T504, A:Q505, A:P506, A:I507, A:R508, A:D509, A:R510, A:K515, A:L516, A:V517, A:C518, A:K519, A:L520, A:P521, A:G522, A:D523, A:F524, A:G525, A:L526, A:V527, A:W538, A:K541, A:H542, A:G543, A:Y544, A:E545, A:T547, A:A549, A:N550, A:Y551, A:T552, A:H553, A:H554, A:W555, A:G556, A:K557, A:V558, A:P559, A:E560, A:W561, A:D562, A:E563, A:N564, A:W565, A:A566, A:E567, A:P568, A:K569, A:I570, A:Q571, A:G573, A:I574, A:N575, A:S576, A:P577, A:G578, A:C579, A:K580, A:D581, A:L582, A:E583, A:T584, A:Q585, A:A586, A:A587, A:S588, A:N589, A:P590, A:Q591, A:S592, A:Q593, A:D594	135	0.71	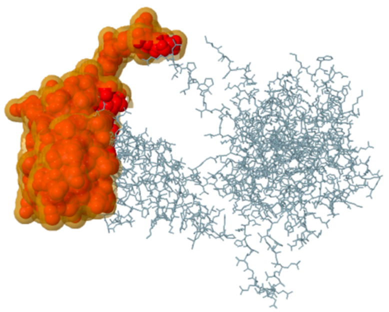
6	A:C32, A:D33, A:N34, A:V35, A:Q36, A:L37, A:N38, A:G39, A:K40, A:D41, A:V42, A:R43, A:W44, A:N45, A:N46, A:Y47, A:T48, A:K49, A:P50, A:I51, A:Q52, A:N53, A:E54, A:E55, A:L56, A:T57, A:S58, A:L59, A:I60, A:R61, A:G62, A:A63, A:Q64, A:T65, A:A66, A:M67, A:D68, A:Q69, A:T70, A:E71, A:E72, A:E73, A:E74, A:D76, A:W77, A:E78, A:S79, A:E80, A:V81, A:D82, A:S83, A:L84, A:A85, A:K86, A:K87, A:Q88, A:Q90, A:T91, A:L95, A:K98, A:E102, A:W146, A:R149, A:Q150, A:N152, A:M153, A:Y154, A:S156, A:R157, A:W158, A:L159, A:V160, A:T161, A:L162, A:C163, A:S164, A:V165, A:N166, A:L167, A:T168, A:P169, A:T170, A:E171, A:K172, A:I173, A:K174, A:L175, A:R176, A:E177, A:I178, A:A179, A:E180, A:D181, A:S182, A:E183, A:W184, A:K218, A:I219, A:V220, A:H221, A:M222, A:T223, A:K224, A:E225, A:S226, A:N239, A:F240, A:M241, A:K242, A:Q244	110	0.704	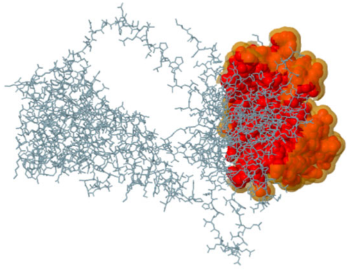
7	A:R191, A:H192, A:K193, A:Q194, A:T195, A:K196, A:K197	7	0.685	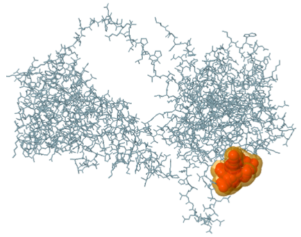

8	A:V105, A:S106, A:K107	3	0.622	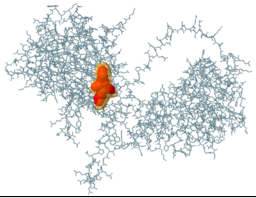
9	A:Y423, A:N424, A:A425, A:A426, A:N427, A:V428, A:N429, A:F430, A:P431, A:N433, A:D468, A:Q469, A:K470, A:G471, A:K472, A:G473, A:S474	17	0.591	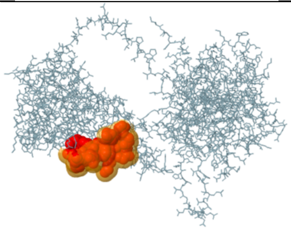
10	A:E661, A:D662, A:F663	3	0.576	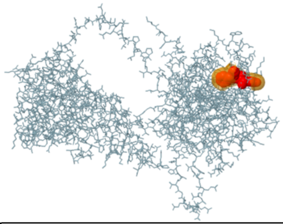
11	A:S2, A:G3, A:N4, A:Q5, A:Y6	5	0.576	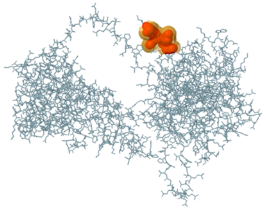
12	A:T655, A:S656, A:E657	3	0.555	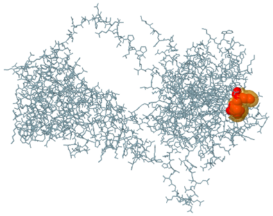

13	A:K124, A:D125, A:Q126	3	0.527	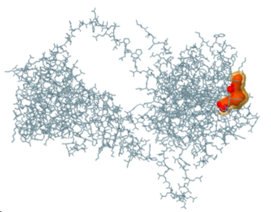

Supplementary Table S6 shows the Predicted conformational (discontinuous) B-cell epitopes of the NS2 protein identified using the ElliPro server and predicted epitopes were mapped onto the three-dimensional structure of NS2 based on structural protrusion analysis

No.	Residues	Number of residues	Score	3D structure with solvent accessibility
1	A:S98, A:S99, A:E100, A:S101, A:R102, A:P103, A:S104, A:N105, A:S106, A:S107, A:D108, A:S109, A:G110, A:R111, A:S112	15	0.836	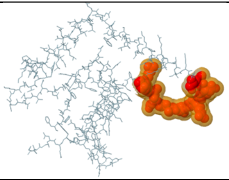
2	A:M1, A:S2, A:G3, A:N4, A:Q5, A:Y6	6	0.798	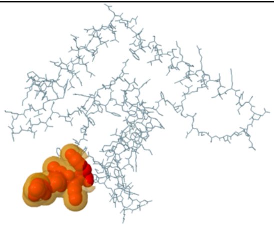
3	A:A125, A:Y126, A:C127, A:R128, A:N129, A:C130, A:K131, A:S132, A:T133, A:I134	10	0.785	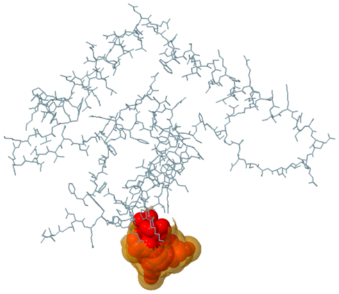
4	A:D92, A:T93, A:S94, A:G95, A:K96	5	0.741	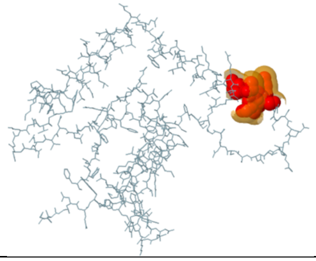

5	A:T56, A:S57, A:R60, A:G61, A:Q63, A:T64, A:D67, A:Q68, A:E70	9	0.694	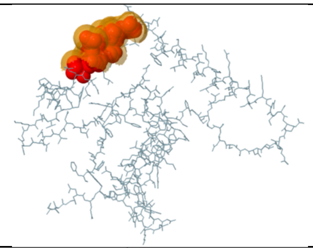
6	A:Q35, A:L36, A:N37, A:G38, A:K39, A:D40, A:R42	7	0.683	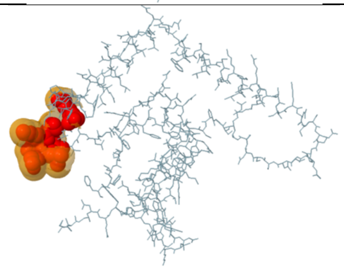
7	A:Y46, A:T47, A:K48, A:P49, A:I50, A:N52, A:E53, A:E54	8	0.674	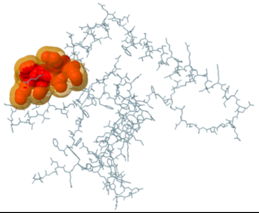
